# Supplementary material for: Bacteriolytic Potential of Enterococcus Phage iF6 Isolated from “Sextaphag®” Therapeutic Phage Cocktail and Properties of Its Endolysins, Gp82 and Gp84
Source: Viruses. 2023 Mar 16;15(3):767. doi: 10.3390/v15030767 (PMC10054541; doi:10.3390/v15030767)
Supplement: Supplementary file 1 [file viruses-15-00767-s001.zip › viruses-2101881-supplementary.pdf]

Bacteriolytic Potential of *Enterococcus* Phage iF6 Isolated from "Sextaphag®" Therapeutic Phage Cocktail and Properties of its Endolysins, Gp82 and Gp84

Rustam M. Buzikov [1,*,†], Olesya A. Kazantseva [1,*,†], Emma G. Piligrimova [1], Natalya A. Ryabova [1,2] and Andrey M. Shadrin [1,*]

[1] Laboratory of Bacteriophage Biology, G. K. Skryabin Institute of Biochemistry and Physiology of Microorganisms, Pushchino Scientific Center for Biological Research of the Russian Academy of Sciences, Federal Research Center, 142290 Pushchino, Russia;
[2] Institute of Protein Research RAS, 142290 Pushchino, Russia
[*] Correspondence: a87h5n1@gmail.com (R.M.B), olesyakazantseva@bk.ru (O.A.K); andrey2010s@gmail.com (A.M.S.)
[†] These authors contributed equally to this work.

Supplementary Information:

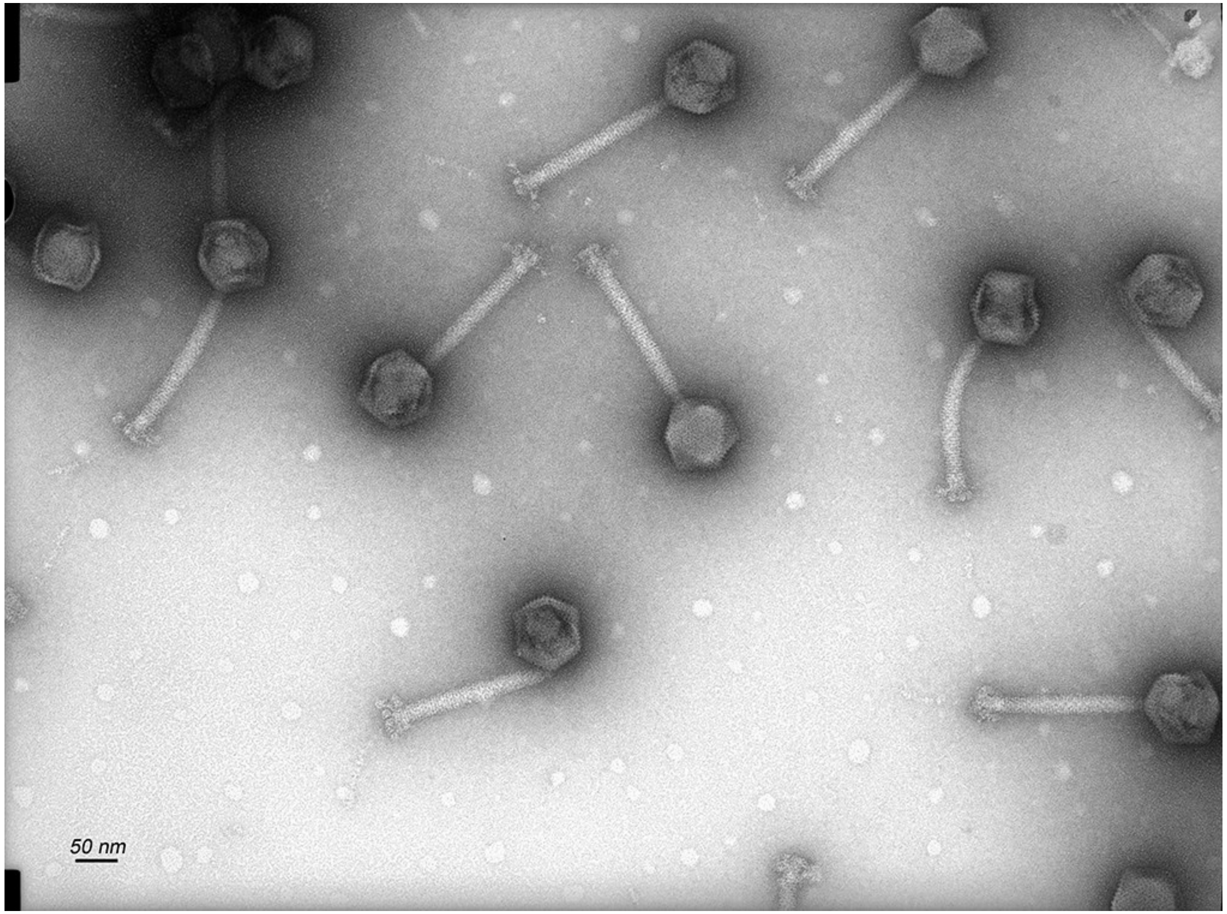

Figure S1. Transmission electron microscopy of *Enterococcus* phage iF6. This is the original TEM micrograph used to generate Figure 1 in the main text. The image was taken using a JEM-100 (JEOL, Japan) transmission electron microscope at 80 kV accelerating voltage on Kodak film SO-163 (Kodak, Cat. # 74144, Hatfield, PA, USA).

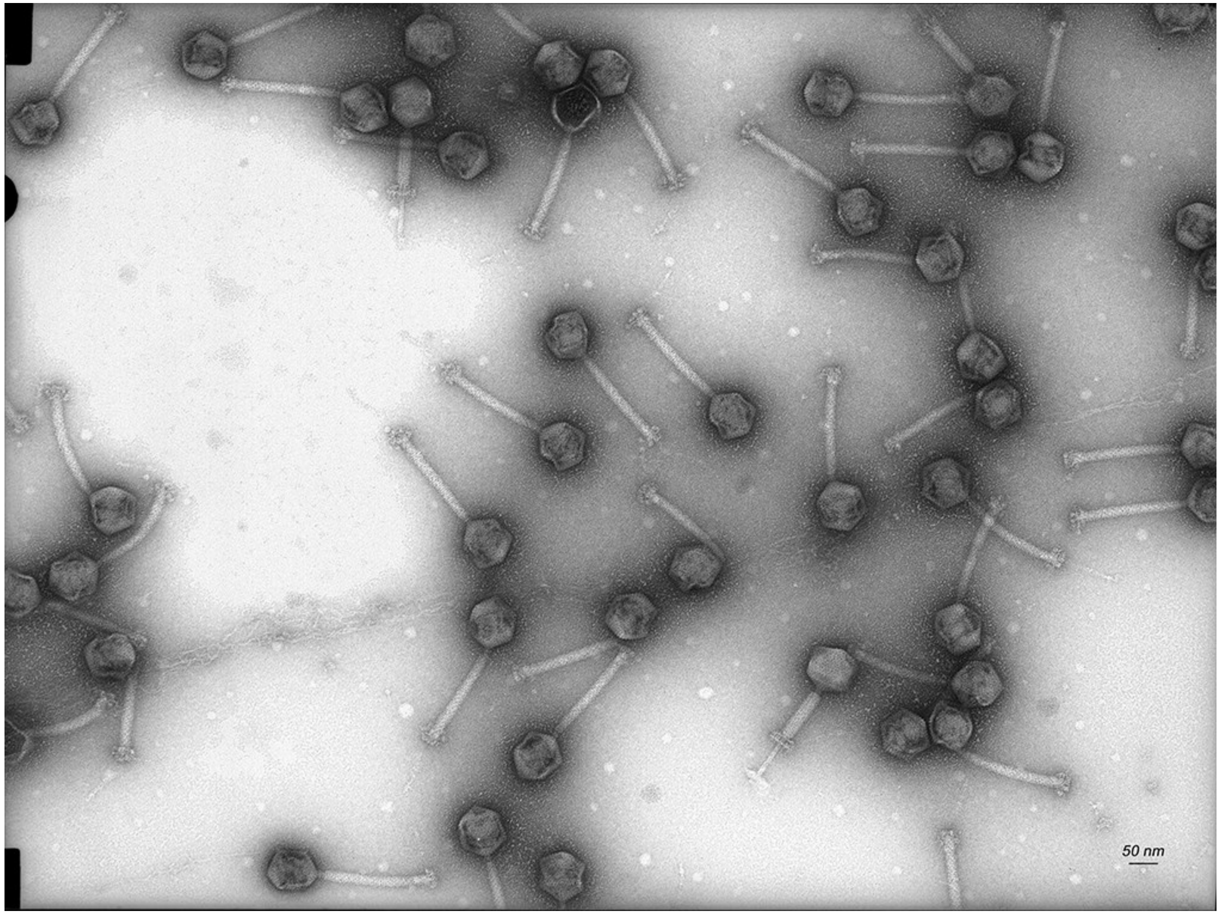

Figure S2. Transmission electron microscopy of *Enterococcus* phage iF6. This is the original TEM micrograph used to generate Figure 1 in the main text. The image was taken using a JEM-100 (JEOL, Japan) transmission electron microscope at 80 kV accelerating voltage on Kodak film SO-163 (Kodak, Cat. # 74144, Hatfield, PA, USA).

Table S1. The host range of the *Enterococcus* phage iF6 and the lytic spectrum of its endolysins determined for 26 *Enterococcus* strains and 3 strains from other genera of bacteria.

No.	Species	Strain	Source*	Cell lysis by:		
				iF6 Phage**	HU-Gp82 endolysin***	HU-Gp84 endolysins***
1	*E. avium*	B-1673	VKM	-	+	+
2	*E. durans*	B-603	VKM	-	++	++
3	*E. durans*	B-8257	VKPM	-	-	-
4	*E. durans*	B-11854	VKPM	-	-	++
5	*E. hirae*	B-12152	VKPM	-	-	+
6	*E. faecalis*	B-12629	VKPM	-	-	+
7	*E. faecalis*	B-4426	VKPM	-	-	+
8	*E. faecalis*	B-4053	VKPM	-	-	++
9	*E. faecium*	FS86	Laboratory collection	+	++	++
10	*E. faecium*	B-2579	VKPM	-	-	+
11	*E. faecium*	B-2990	VKPM	+	+	+
12	*E. faecium*	B-3490	VKPM	+	-	-
13	*E. faecium*	B-3491	VKPM	-	-	+
14	*E. faecium*	B-4054	VKPM	-	-	+
15	*E. faecium*	B-4489	VKPM	-	+	+
16	*E. faecium*	B-4991	VKPM	+	-	-
17	*E. faecium*	B-5000	VKPM	-	-	++
18	*E. faecium*	B-8551	VKPM	+	+	++
19	*E. faecium*	B-12648	VKPM	-	-	-
20	*E. thailandicus*	B-10684	VKPM	+	-	++
21	*E. sp*	B-1578	VKM	+	+	+
22	*E. sp*	B-1944	VKM	+	+	+
23	*E. sp*	B-3371	VKPM	-	-	-
24	*E. sp*	B-8711	VKPM	-	-	+
25	*E. sp*	B-8712	VKPM	-	+	++

26	*E. sp*	B-8713	VKPM	+	-	-
27	*E. coli*	XL1blue	Laboratory collection	-	-	-
28	*P. syringae*	B-268	BIM	-	-	-
29	*B. cereus*	B-370	VKM	-	-	-

* Source abbreviations: VKM: All-Russian Collection of Microorganisms; VKPM: National Bioresource Center – All-Russian collection of industrial microorganisms; BIM – the Belarusian collection of non-pathogenic microorganisms (BIM) of the Institute of Microbiology, National Academy of Sciences.

** Symbols for the host range of *Enterococcus* phage iF6: “+” – strains susceptible to the iF6 phage; “-“ – strains resistant to the iF6 phage.

*** Symbols for the lytic spectrum of the HU-Gp82 and HU-Gp84 endolysins: “++” – strains inhibited by a low dose of endolysins (15 µg/mL and 11 µg/mL, respectively); “+” – strains inhibited by a higher dose of endolysins (30 µg/mL and 22 µg/mL, respectively); “-“ – strains resistant to endolysins.

Table S2. Annotation of *Enterococcus* phage iF6.

ORF№	Start codon	Stop codon	Strand	Blast results		Conserved domains, Blast		Hhpred results	Annotation
				Name	E-val	Name, (region)	E-val	(Prob./E-val)	
1	497	652	+	hp	hp	-	-	('-', '-')	hp
2	703	969	+	hp	hp	-	-	d1vqqa2; d.175.1.1 (A:139-327) Penicillin binding protein 2a (PBP2A), middle domain {*Staphylococcus aureus* [TaxId: 1280]} (64.56/4.10e+00)	hp
3	986	1228	+	hp	hp	-	-	PF16112.6; DUF4830; Domain of unknown function (DUF4830) (95.11/1.30e-02)	hp
4	1245	1565	+	hp	hp	-	-	PF03871.15; RNA_pol_Rpb5_N; RNA polymerase Rpb5, N-terminal domain (88.98/2.60e+00)	hp
5	1639	1779	+	hp	hp	-	-	('-', '-')	hp
6	1869	2012	+	hp	hp	-	-	PF13035.7; DUF3896; Protein of unknown function (DUF3896) (87.29/2.50e+00)	hp
7	2417	2205	-	hp	hp	-	-	('-', '-')	hp
8	2745	2473	-	hp	hp	-	-	PF03886.14; ABC_trans_aux; ABC-type transport auxiliary	hp

								lipoprotein component (83.38/4.20e+00)	
9	3173	2763	-	hp	hp	-	-	('-', '-')	hp
10	3412	3197	-	hp	hp	-	-	PF06785.12; UPF0242; Uncharacterised protein family (UPF0242) N-terminus (87.68/2.70e+00)	hp
11	3640	3413	-	hp	hp	-	-	('-', '-')	hp
12	3875	3642	-	hp	hp	-	-	KOG2983; Uncharacterized conserved protein [Function unknown] (73.96/4.50e+00)	hp
13	4267	3962	-	hp	hp	-	-	PF07862.12; Nif11; Nif11 domain (86.96/1.90e+00)	hp
14	4476	4279	-	hp	hp	-	-	('-', '-')	hp
15	4640	4476	-	hp	hp	-	-	COG4416; Com; Mu-like prophage FluMu protein Com [Mobilome: prophages, transposons]. (83.46/4.70e-01)	hp
16	5052	4630	-	hp	hp	-	-	('-', '-')	hp
17	5137	5045	-	protein RtcB [*Enterococcus* phage vB_OCPT_Ben]<br><br>RtcB protein [*Enterococcus* phage PEf771]<br><br>protein RtcB [*Enterococcus* phage EFDG1]	5.0e-12<br><br>5.0e-12<br><br>5.0e-12	-	-	('-', '-')	hp

18	5318	5130	-	hp	hp	-	-	PF11460.9; DUF3007; Protein of unknown function (DUF3007) (79.24/2.40e+00)	hp
19	6150	5407	-	hp	hp	-	-	COG1996; RPC10; DNA-directed RNA polymerase, subunit RPC12/RpoP, contains C4-type Zn-finger [Transcription]. (86.18/7.60e-01)	hp
20	7082	6228	-	hp	hp	-	-	PF09954.10; DUF2188; Uncharacterized protein conserved in bacteria (DUF2188) (80.11/5.40e+00)	hp
21	7393	7079	-	NAD-dependent DNA ligase [*Streptococcus* phage Dp-1]<br><br>gp102 [*Rhodococcus* phage ReqiPepy6]<br><br>DNA ligase [*Clostridium* phage phi8074-B1]	7.0e-10<br><br>5.0e-09<br><br>3.0e-08	ligA (17-52)	1.24e-04	d1dgsa3; d.142.2.2 (A:1-314) Adenylation domain of NAD+-dependent DNA ligase {*Thermus filiformis* [TaxId: 276]} (99.79/5.90e-19)<br><br>PF01653.19; DNA_ligase_aden; NAD-dependent DNA ligase adenylation domain (99.78/8.20e-19)<br><br>d1ta8a_; d.142.2.2 (A:) Adenylation domain of NAD+-dependent DNA ligase {*Enterococcus faecalis* [TaxId: 1351]} (99.78/8.50e-19)	NAD-dependent DNA ligase adenylation domain containing protein
22	7878	7396	-	hp	hp	HTH_XRE (106-154)	4.68e-12	COG1395; COG1395; Predicted transcriptional regulator [Transcription]. (99.41/4.50e-13)	DNA-binding protein

								COG5606; COG5606; Predicted DNA-binding protein, XRE-type HTH domain [General function prediction only]. (98.67/1.20e-06)<br><br>KOG3398; Transcription factor MBF1 [Transcription] (98.55/2.60e-06)	
23	8274	8044	-	hp	hp	-	-	PF14353.7; CpXC; CpXC protein(98.36/2.80e-06)<br><br>PF11520.9; Cren7; Chromatin protein Cren7(98.29/5.60e-06)<br><br>PF09538.11; FYDLN_acid; Protein of unknown function (FYDLN_acid) (97.98/1.40e-05)	Zn-binding domain containing protein
24	8582	8286	-	hp	hp	-	-	PF17399.3; DUF5405; Domain of unknown function (DUF5405) (99.57/4.20e-14)	DUF5405-containing protein
25	9028	8582	-	hp	hp	-	-	('-', '-')	hp
26	9614	9216	-	hp	hp	-	-	d1twfi1; g.41.3.1 (I:1-49) RBP9 subunit of RNA polymerase II {Baker's yeast (*Saccharomyces cerevisiae*) [TaxId: 4932]} (91.09/1.80e-01)	hp
27	9870	9607	-	hp	hp	-	-	('-', '-')	hp
28	11580	9946	-	hp	hp	-	-	('-', '-')	hp
29	11916	11596	-	hp	hp	-	-	('-', '-')	hp

30	12730	11990	-	Serine/threonine protein phosphatase (EC [*Enterococcus* phage 163]<br><br>serine/threonine protein phosphatase [*Enterococcus* phage EFDG1]<br><br>serine/threonine protein phosphatase [*Enterococcus* phage EfV12-phi1]	1.0e-162<br><br>3.0e-159<br><br>5.0e-159	MPP_PPP_family (8-226)<br><br>Metallophos (8-192)<br><br>bacter_Pnkp (8-76)	1.24e-23<br><br>2.64e-09<br><br>1.82e-07	cd07413; MPP_PA3087; *Pseudomonas aeruginosa* PA3087 and related proteins, metallophosphatase domain.(99.9/8.00e-22)<br><br>cd07421; MPP_Rhilphs; Rhilph phosphatases, metallophosphatase domain.(99.86/1.70e-20)<br><br>d1g5ba_; d.159.1.3 (A:) lambda ser/thr protein phosphatase {Bacteriophage lambda [TaxId: 10710]}(99.86/2.20e-20)	serine/threonine protein phosphatase
31	12987	12730	-	hp	hp	-	-	('-', '-')	hp
32	13592	12984	-	putative metallo-dependent phosphatase 1 [*Enterococcus* phage EFP01]<br><br>Phosphoesterase [*Enterococcus* phage 163]<br><br>phosphoesterase [*Enterococcus* phage vB_OCPT_Ben]	5.0e-135<br><br>1.0e-134<br><br>1.0e-133	MPP_AQ1575 (4-181)<br><br>COG4186 (2-159)<br><br>yfcE (1-145)	2.90e-34<br><br>3.73e-24<br><br>5.53e-05	COG4186; COG4186; Calcineurin-like phosphoesterase superfamily protein [General function prediction only]. (99.87/2.20e-20)<br><br>d1xm7a1; d.159.1.8 (A:1-186) hp aq_1666 {*Aquifex aeolicus* [TaxId: 63363]} (99.81/5.20e-19)<br><br>cd07390; MPP_AQ1575; *Aquifex aeolicus* AQ1575 and related proteins,	calcineurin-like phosphoesterase

								metallophosphatase domain. (99.8/1.40e-18)	
33	14164	13589	-	phosphoesterase [*Enterococcus* phage PEf771]<br><br>phosphohydrolase [*Enterococcus* phage EFDG1]<br><br>putative phosphoesterase or phosphohydrolase [*Enterococcus* phage EfsSzw-1]	9.0e-124<br><br>1.0e-123<br><br>2.0e-122	MPP_AQ1575 (4-166)<br><br>COG4186 (1-165)<br><br>Metallophos_2 (32-139)	9.62e-28<br><br>2.85e-25<br><br>3.26e-06	COG4186; COG4186; Calcineurin-like phosphoesterase superfamily protein [General function prediction only]. (99.84/9.20e-19)<br><br>cd07390; MPP_AQ1575; *Aquifex aeolicus* AQ1575 and related proteins, metallophosphatase domain. (99.8/1.40e-18)<br><br>d1xm7a1; d.159.1.8 (A:1-186) hp aq_1666 {*Aquifex aeolicus* [TaxId: 63363]} (99.76/2.40e-17)	calcineurin-like phosphoesterase
34	14695	14165	-	exonuclease [*Enterococcus* phage EFP01]<br><br>exonuclease [*Enterococcus* phage PEf771]<br><br>gp128 [*Bacillus* virus G]	7.0e-117<br><br>7.0e-117<br><br>3.0e-55	DUF5051 (1-164)	3.49e-09	COG2176; PolC; DNA polymerase III, alpha subunit (gram-positive type) [Replication, recombination and repair]. (99.85/6.20e-20)<br><br>KOG0542; Predicted exonuclease [Replication, recombination and repair] (99.83/2.10e-18)<br><br>KOG0304; mRNA deadenylase subunit [RNA processing and modification] (99.76/6.00e-17)	3' exoribonuclease family protein

								KOG3242 Oligoribonuclease (3'5' exoribonuclease) [RNA processing and modification] (99.71/1.1e-15)	
35	15076	14726	-	hp EFV12PHI1_12<br><br>Phage protein [*Enterococcus* phage 163]<br><br>gp107 [*Lactococcus* virus KSY1]	8.0e-73<br><br>6.0e-70<br><br>8.0e-18	DUF3307 (8-116)	1.33e-04	PF11750.9; DUF3307; Protein of unknown function (DUF3307) (99.93/1.60e-24)	DUF3307-containing protein
36	15642	15073	-	hp	hp	-	-	('-', '-')	hp
37	15899	15639	-	hp	hp	-	-	('-', '-')	hp
38	16166	15900	-	Phage-related protein [*Clostridium* phage phiCD111]<br><br>Phage protein [*Streptococcus* phage IC1]	6.0e-17<br><br>2.0e-15	-	-	PF14083.7; PGDYG; PGDYG protein (99.54/8.10e-14)<br><br>PF14083.7; PGDYG; PGDYG protein (98.68/5.50e-08)	PGDYG motif containing protein
39	16436	16260	-	hp	hp	-	-	d1vk6a4; g.41.14.1 (A:97-125) NADH pyrophosphatase intervening domain {*Escherichia coli* [TaxId: 562]} (82.77/1.50e+00)	hp
40	17013	16450	-	hp	hp	-	-	('-', '-')	hp
41	17107	17015	-	hp	hp	-	-	d1dgsa1; a.60.2.2 (A:401-581) NAD+-dependent DNA ligase, domain 3 {*Thermus filiformis* [TaxId: 276]} (96.51/2.20e-03)	hp

								d1yuza2; g.41.5.1 (A:167-202) Nigerythrin, C-terminal domain {*Desulfovibrio vulgaris* [TaxId: 881]} (95.48/1.10e-02)<br><br>COG1592; YotD; Rubrerythrin [Energy production and conversion]. (94.84/1.90e-02)	
42	17339	17181	-	hp	hp	-	-	('-', '-')	hp
43	18552	17428	-	transposase [*Geobacillus* virus E3]<br><br>gp191 [*Brochothrix* phage A9]<br><br>transposase [*Paenibacillus* phage Unity]	4.0e-153<br><br>7.0e-132<br><br>2.0e-113	InsQ (3-368)<br><br>OrfB_IS605 (163-276)<br><br>tspaseT_teng_C (242-316)	1.26e-61<br><br>1.22e-27<br><br>3.78e-10	COG0675; InsQ; Transposase [Mobilome: prophages, transposons]. (100.0/1.50e-35)<br><br>PF18516.2; RuvC_1; RuvC nuclease domain (99.69/6.50e-16)<br><br>PF01385.20; OrfB_IS605; Probable transposase (99.0/2.30e-08)	transposase
44	19384	18770	-	ribonucleotide reductase of class III (anaerobic), activating protein [*Enterococcus* phage EFDG1]<br><br>ribonucleotide reductase of class III (anaerobic), activating protein [*Enterococcus* phage vB_OCPT_Ben]	2.0e-136<br><br>4.0e-136	Fer4_12 (27-169)<br><br>NrdG (17-171)	2.00e-67<br><br>2.20e-61	cd17825; Radical_SAM_MoaA; Radical_SAM. (99.82/9.60e-20)<br><br>cd17853; Radical_SAM; Radical_SAM (99.81/2.00e-19	ribonucleotide reductase of class III (anaerobic)

				ribonucleotide reductase of class III (anaerobic), activating protein [*Enterococcus* phage EfV12-phi1]	2.0e-135				
45	19670	19377	-	hp	hp	-	-	PF18450.2; zf_C2H2_6; Zinc Finger domain (95.73/3.50e-03) d1fu9a1; g.37.1.2 (A:3-36) (94.57/1.30e-02)	hp
46	20632	19670	-	thymidylate synthase [*Enterococcus* phage EFDG1]<br><br>thymidylate synthase [*Enterococcus* phage vB_OCPT_Ben]<br><br>thymidylate synthase [*Enterococcus* phage PEf771]	0.0e+00<br><br>0.0e+00<br><br>0.0e+00	Thymidylat_synt (53-320)<br><br>TS_Pyrimidine_HMase (6-264)<br><br>thym_sym (53-308)	8.19e-82<br><br>1.76e-52<br><br>1.80e-40	d1bkpa_; d.117.1.1 (A:) Thymidylate synthase {*Bacillus subtilis* [TaxId: 1423]} (100/3.20e-62)<br><br>d1j3kc_; d.117.1.1 (C:) Bifunctional enzyme dihydrofolate reductase-thymidylate synthase, TS domain {Malaria parasite (*Plasmodium falciparum*) [TaxId: 5833]} (100/2.30e-60)<br><br>d1tisa_; d.117.1.1 (A:) Thymidylate synthase {Bacteriophage T4 [TaxId: 10665]} (100/7.50e-60)	thymidylate synthase
47	21271	20648	-	hp	hp	-	-	PF10892.9; DUF2688; Protein of unknown function (DUF2688) (93.14/5.20e-02)	hp
48	22022	21273	-	hp	hp	-	-	('-', '-')	hp

49	22563	22096	-	hp	hp	-	-	d2fvva1 d.113.1.1 (A:8-142) Diphosphoinositol polyphosphate phosphohydrolase {Human (Homo sapiens) [TaxId: 9606]} (99.56/7.2e-13)<br><br>cd18873 Nudix_ADPR_NadM_NrtR; Nudix hydrolase subfamily 7. (99.49/2e-12)<br><br>Nucleoside triphosphate pyrophosphorylase (MutT) {*Escherichia coli* K-12 [TaxId: 83333]} (99.47/1e-11)	nucleoside triphosphate pyrophosphorylase
49	22563	22096	-	hp	hp	-	-	d2fvva1 d.113.1.1 (A:8-142) Diphosphoinositol polyphosphate phosphohydrolase {Human (Homo sapiens) [TaxId: 9606]} (99.56/7.2e-13)<br><br>cd18873 Nudix_ADPR_NadM_NrtR; Nudix hydrolase subfamily 7. (99.49/2e-12)<br><br>Nucleoside triphosphate pyrophosphorylase (MutT) {*Escherichia coli* K-12 [TaxId: 83333]} (99.47/1e-11)	nucleoside triphosphate pyrophosphorylase

50	23315	22578	-	hp	hp	NMN_transporter (44-232)	4.24e-10	COG3201; PnuC; Nicotinamide riboside transporter PnuC [Coenzyme transport and metabolism]. (100.0/2.40e-36)<br><br>PF04973.13; NMN_transporter; Nicotinamide mononucleotide transporter (100.0/1.70e-32)<br><br>PF04973.13; NMN_transporter; Nicotinamide mononucleotide transporter (98.69/1.50e-07)	nicotinamide mononucleotide transporter
51	23679	23317	-	hp	hp	-	-	PF03698.14; UPF0180; Uncharacterised protein family (UPF0180) (82.59/2.70e+00)	hp
52	24806	23679	-	nicotinamide-nucleotide adenylyltransferase, NadR family/ ribosylnicotinamide kinase [*Enterococcus* phage vB_OCPT_Ben]<br><br>nicotinamide-nucleotide adenylyltransferase, NadR family / ribosylnicotinamide kinase [*Enterococcus* phage EfV12-phi1]<br><br>nicotinamide-nucleotide adenylyltransferase, NadR family /	0.0e+00<br><br>0.0e+00<br><br>0.0e+00	PRK08099 (1-355)<br><br>NadR3 (171-353)<br><br>NMNAT_NadR (5-174)	1.14e-164<br><br>3.41e-65<br><br>9.06e-42	d1lw7a1; c.26.1.3 (A:57-219) Transcriptional regulator NadR, NMN-adenylyltransferase domain {*Haemophilus influenzae* [TaxId: 727]} (99.85/7.80e-20)<br><br>cd02167; NMNAT_NadR; Nicotinamide/nicotinate mononucleotide adenylyltransferase of bifunctional NadR-like proteins. (99.78/2.70e-18)<br><br>KOG2803; Choline phosphate cytidylyltransferase/Predicted CDP-ethanolamine synthase	NadR transcriptional regulator /nicotinamide-nucleotide adenylyltransferase, NadR family/ ribosylnicotinamide kinase

				ribosylnicotinamide kinase [*Enterococcus* phage EFDG1]				[Lipid transport and metabolism] (99.63/5.80e-16)	
53	25254	24892	-	HAD-like domain containing protein [*Enterococcus* phage EfsSzw-1]<br><br>gp153 [*Listeria* virus P100]<br><br>HAD-like domain containing protein [*Listeria* phage LMTA-94]	2.0e-57<br><br>3.0e-21<br><br>1.0e-20	Cof (8-78)	6.38e-03	d2obba1; c.108.1.25 (A:1-122) hp BT0820 {*Bacteroides thetaiotaomicron* [TaxId: 818]} (99.76/9.10e-17)<br><br>PF06189.13; 5-nucleotidase; 5'-nucleotidase (99.3/9.30e-12)<br><br>COG5663 YqfW; Uncharacterized protein, HAD superfamily [General function prediction only]. (99.11/2.9e-10)<br><br>d1ltqa1; c.108.1.9 (A:153-301) Polynucleotide kinase, phosphatase domain {Bacteriophage T4 [TaxId: 10665]} (99.21/1.10e-10)<br><br>PF12689.8; Acid_PPase; Acid Phosphatase (98.93/6.2e-9)	HAD-like family phosphatase
54	25588	25349	-	putative ribonucleotide reductase [*Enterococcus* phage EFP01]<br><br>glutaredoxin-like protein [*Enterococcus* phage EfV12-phi1]	5.0e-48<br><br>4.0e-45	GlrX_NrdH (4-74)<br><br>Glutaredoxin (4-48)	6.89e-18<br><br>9.46e-12<br><br>1.50e-09	d1r7ha_; c.47.1.1 (A:) Glutaredoxin-like NRDH-redoxin {*Corynebacterium ammoniagenes* [TaxId: 1697]} (99.37/9.50e-11)<br><br>d1h75a_; c.47.1.1 (A:) Glutaredoxin-like NRDH-	glutaredoxin-like NRDH-redoxin

				putative ribonucleotide reductase [*Enterococcus* phage EfsSzw-1]	6.0e-44	GrxC (3-76)		redoxin {*Escherichia coli* [TaxId: 562]} (99.36/1.10e-10)<br><br>d1nhoa_; c.47.1.1 (A:) MTH807, thioredoxin/glutaredoxin-like protein {*Methanobacterium thermoautotrophicum* [TaxId: 145262]} (99.15/3.80e-09)	
55	26345	25581	-	Nicotinamide mononucleotide transporter [*Enterococcus* phage 163]<br><br>membrane protein [*Enterococcus* phage EFP01]<br><br>nicotinamide mononucleotide transporter [*Enterococcus* phage EfV12-phi1]<br><br>NrdR-regulated deoxyribonucleotide transporter [*Enterococcus* phage EFDG1]	2.0e-171<br><br>1.0e-170<br><br>9.0e-170<br><br>1.0e-140	NMN_transporter (70-248)<br><br>NMN_trans_PnuC (70-250)	1.59e-20<br><br>8.37e-14	COG3201; PnuC; Nicotinamide riboside transporter PnuC [Coenzyme transport and metabolism]. (100.0/1.20e-34)<br><br>PF04973.13; NMN_transporter; Nicotinamide mononucleotide transporter (99.97/3.40e-30)<br><br>PF04973.13; NMN_transporter; Nicotinamide mononucleotide transporter (98.33/3.00e-06)	PnuC-like nicotinamide mononucleotide transporter
56	27178	26363	-	deoxyguanosine kinase [*Enterococcus* phage PEf771]<br><br>kinase [*Enterococcus* phage EF1]	0.0e+00<br><br>0.0e+00	Dck (1-207)<br><br>dNK (2-193)	4.63e-61<br><br>2.86e-48	cd01673; dNK; Deoxyribonucleoside kinase (dNK) catalyzes the phosphorylation of deoxyribonucleosides to yield corresponding monophosphates (dNMPs). (99.83/1.60e-17)	deoxyribonucleoside kinase

				deoxynucleoside kinase family protein [*Enterococcus* phage EfsSzw-1]	0.0e+00			PF01712.20; dNK; Deoxynucleoside kinase (99.83/1.60e-17)<br><br>COG1428; Dck; Deoxyadenosine/deoxycytidine kinase [Nucleotide transport and metabolism]. (99.83/4.20e-17)	
57	28119	27367	-	-	-	LAGLIDADG_2 (61-201)	3.42e-06	COG3780; COG3780; DNA endonuclease related to intein-encoded endonucleases [Replication, recombination and repair]. (99.87/1.80e-20)<br><br>d1r7ma2; d.95.2.1 (A:121-225) DNA endonuclease I-SceI {Baker's yeast (*Saccharomyces cerevisiae*) [TaxId: 4932]} (99.74/1.90e-17)<br><br>cd09958; LAGLIDADG_1_child_11_child_1 LAGLIDADG endonuclease. LAGLIDADG endonuclease (99.67/1.30e-15)	DNA endonuclease
58	30475	28370	-	ribonucleotide reductase of class III (anaerobic), large subunit [*Enterococcus* phage EfV12-phi1]	0.0e+00<br><br>0.0e+00	PRK09263 (14-698)<br><br>NRDD	0e+00<br><br>0e+00	d1hk8a_; c.7.1.3 (A:) Class III anaerobic ribonucleotide reductase NRDD subunit {Bacteriophage T4 [TaxId: 10665]} (100.0/2.80e-91)	class III ribonucleotide reductase

				ribonucleotide reductase of class III (anaerobic), large subunit [*Enterococcus* phage EFDG1]<br><br>Ribonucleotide reductase of class III (anaerobic), large subunit (EC [*Enterococcus* phage 163]	0.0e+00	(131-698)		PF13597.7; NRDD; Anaerobic ribonucleoside-triphosphate reductase (100.0/3.40e-83)<br><br>COG1328; NrdD; Anaerobic ribonucleoside-triphosphate reductase [Nucleotide transport and metabolism]. (100.0/9.30e-82)	
59	30895	30626	-	hp	hp	-	-	PF13113.7; DUF3970; Protein of unknown function (DUF3970) (80.08/2.20e+00)<br><br>PF10369.10; ALS_ss_C; Small subunit of acetolactate synthase (80.52/2.6)	hp
60	31737	30907	-	ParBc domain protein [*Streptococcus* phage phiARI0831b]<br><br>ParB-like nuclease domain containing protein [uncultured *Mediterranean* phage uvMED]<br><br>ParB-like nuclease domain containing protein [uncultured *Mediterranean* phage uvMED]	2.0e-14<br><br>1.0e-11<br><br>7.0e-09	pNOB8_ParB_N_like (25-83)<br><br>ParB (29-90)	6.29e-14<br><br>1.78e-06	COG1475; Spo0J; Chromosome segregation protein Spo0J, contains ParB-like nuclease domain [Cell cycle control, cell division, chromosome partitioning]. (99.37/9.30e-12)<br><br>d1vz0a2; d.268.1.1 (A:23-115) Putative partitioning protein ParB/Spo0J {*Thermus thermophilus* [TaxId: 274]} (99.19/2.10e-10)<br><br>cd16402; ParB_N_like_MT; ParB N-terminal-like domain, some attached to C-terminal S-	ParB-like nuclease domain containing protein

								adenosylmethionine-dependent methyltransferase domain. (98.98/7.30e-09)	
61	32061	31756	-	putative scaffolding protein [*Enterococcus* phage EFDG1]<br><br>hp [*Enterococcus* phage 163]	3.0e-60<br><br>1.0e-59	YnbB (11-85)	3.21e-03	COG5377; COG5377; Phage-related protein, predicted endonuclease [Mobilome: prophages, transposons]. (99.44/3.80e-12)<br><br>PF05565.12; Sipho_Gp157; Siphovirus Gp157 (96.4/0.1)	putative endonuclease
62	32377	32162	-	putative transcriptional regulator [*Enterococcus* phage EfsSzw-1]<br><br>repressor [*Enterococcus* phage vB_EfaM_Ef2.1]<br><br>Phage protein [*Enterococcus* phage 156]	1.0e-40<br><br>5.0e-19<br><br>1.0e-18	HTH_XRE (10-64)<br><br>HipB (10-71)	1.46e-11<br><br>8.85e-06	COG5606; COG5606; Predicted DNA-binding protein, XRE-type HTH domain [General function prediction only]. (98.76/3.10e-07)<br><br>d1b0na2; a.35.1.3 (A:1-68) SinR repressor, DNA-binding domain {*Bacillus subtilis* [TaxId: 1423]} (98.7/3.70e-07)<br><br>COG1395 COG1395; Predicted transcriptional regulator [Transcription]. (98.67/e-06)	putative transcriptional regulator

63	33968	32436	-	DUF2828 domain-containing protein [*Enterococcus* phage EfsSzw-1]<br><br>Phage protein [*Enterococcus* phage 163]<br><br>DUF2828 domain-containing protein [*Listeria* phage LP-048]	0.0e+00<br><br>0.0e+00<br><br>3.0e-121	DUF2828 (16-491)	6.55e-73	PF11443.9; DUF2828; Domain of unknown function (DUF2828) (100.0/1.40e-84)<br><br>KOG4465; Uncharacterized conserved protein [Function unknown] (99.84/1.60e-19)<br><br>d1yvra2; c.62.1.5 (A:364-537) 60-kda SS-A/Ro ribonucleoprotein, RoRNP {African clawed frog (*Xenopus laevis*) [TaxId: 8355]} (98.99/9.40e-08)	DUF2828 domain-containing protein
64	35020	34796	-	hp	hp	-	-	('-', '-')	hp
65	37328	36960	-	hp	hp	-	-	('-', '-')	hp
66	37902	37720	-	hp	hp	-	-	COG3112; YacL; Uncharacterized protein YacL, UPF0231 family [Function unknown]. (77.62/4.60e+00)	hp
67	39444	39223	-	hp	hp	-	-	PF18401.2; Thioredoxin_13; Thioredoxin-like domain (71.23/7.60e+00)	hp
68	39704	39453	-	hp	hp	-	-	('-', '-')	hp
69	44975	44637	-	hp [*Enterococcus* phage 163]<br><br>structural protein [*Enterococcus* phage ECP3]	1.0e-70<br><br>3.0e-11	-	-	PF06289.12; FlbD; Flagellar and Swarming motility proteins (88.98/7.60e-01)	hp

				putative structural protein [*Enterococcus* phage phiM1EF22]<br><br>structural protein [*Enterococcus* phage EF24C]	2.0e-10<br><br>2.0e-10				
70	45377	45030	-	hp EFP01_107 [*Enterococcus* phage EFP01]<br><br>gp67 [*Listeria* virus A511]<br><br>putative membrane bound protein [*Staphylococcus* phage f2b1]<br><br>putative membrane protein [*Bacillus* phage Bcp1]	2.0e-72<br><br>1.0e-22<br><br>4.0e-16<br><br>5.0e-12	PHA02414 (1-114)	7.01e-50	PF10779.10; XhlA; Haemolysin XhlA (96.23/1.10e-01)	putative haemolysin XhlA
71	45674	45417	-	hp	hp	-	-	PF04678.14; MCU; Mitochondrial calcium uniporter (84.73/4.70e+00)	hp
72	45793	46062	+	hp	hp	-	-	COG3771; YciS; Uncharacterized membrane protein YciS, DUF1049 family [Function unknown]. (99.4/6.80e-12)<br><br>COG5416; YrvD; Uncharacterized integral	putative membrane protein

								membrane protein [Function unknown]. (99.26/3.10e-11)<br><br>PF06305.12; LapA_dom; Lipopolysaccharide assembly protein A domain (98.99/1.60e-09)	
73	46082	46360	+	gp71 [*Listeria* virus A511]<br><br>membrane protein [*Staphylococcus* phage phiIPLA-C1C]<br><br>Dmd [*Staphylococcus* phage JD007]	6.0e-16<br><br>2.0e-15<br><br>8.0e-10	-	-	PF10013.10; DUF2256; Uncharacterized protein conserved in bacteria (DUF2256) (90.96/1.30e-01)	hp
74	46365	46793	+	Phage protein [*Enterococcus* phage 156]<br><br>gp72 [*Listeria* virus A511]<br><br>ORF092 [*Staphylococcus* virus Twort]	4.0e-58<br><br>1.0e-19<br><br>40e-15	-	-	('-', '-')	hp
75	46793	47080	+	terminase large subunit [*Enterococcus* phage EFP01]<br><br>terminase large subunit [*Enterococcus* phage EfV12-phi1]	6.0e-61<br><br>1.0e-54<br><br>1.0e-54	-	-	COG5525; YbcX; Phage terminase, large subunit GpA [Mobilome: prophages, transposons]. (67.42/2.30e+00)<br><br>PF18305.2; DNA_pol_A_exoN; 3' to 5' exonuclease C-terminal domain (60.85/2.6)	putative terminase, large subunit

				terminase large subunit [*Enterococcus* phage EfsSzw-1]				PF03354.16; Terminase_1; Phage Terminase (63.85/5.3)	
76	47349	48269	+	hydrolase [*Lactobacillus* phage Iacchus]<br><br>ORF031 [*Staphylococcus* virus Twort]<br><br>hydrolase [*Staphylococcus* phage vB_SsapH-Golestan101-M]<br><br>homing endonuclease [*Lactobacillus* phage 3-521] 3.0e-82	4.0e-90<br><br>4.0e-88<br><br>3.0e-80<br><br>2.0e-70	-	-	d1vsra_; c.52.1.15 (A:) Very short patch repair (VSR) endonuclease {*Escherichia coli* [TaxId: 562]} (98.32/2.10e-05)<br><br>PF14311.7; DUF4379; Probable Zinc-ribbon domain (97.68/1.90e-05)<br><br>PF05265.14; DUF723; Protein of unknown function (DUF723) (98.12/1.80e-05)	VSR endonuclease
77	48715	49674	+	Phage protein [*Enterococcus* phage 156]<br><br>VSR homing endonuclease [*Staphylococcus* phage phiIPLA-RODI]<br><br>group I intron protein [*Staphylococcus* phage vB_SauM_Romulus]	0.0e+00<br><br>6.0e-47<br><br>1.0e-40	DUF559 (226-307)<br><br>Vsr (195-290)<br><br>YcjD(226-307)	6.33e-06<br><br>1.23e-04<br><br>3.30e-04	d1vsra_; c.52.1.15 (A:) Very short patch repair (VSR) endonuclease {*Escherichia coli* [TaxId: 562]} (98.96/1.70e-08)<br><br>COG3727; Vsr; G:T-mismatch repair DNA endonuclease, very short patch repair protein [Replication, recombination and repair]. (98.73/3.30e-07)<br><br>PF14311.7; DUF4379; Probable Zinc-ribbon domain (98.25/8.50e-07)	VSR endonuclease

78	49705	51210	+	terminase large subunit [*Enterococcus* phage vB_OCPT_Ben]<br><br>terminase large subunit [*Enterococcus* phage EFDG1]<br><br>Phage terminase, large subunit [*Enterococcus* phage 163]	0.0e+00<br><br>0.0e+00<br><br>0.0e+00	Terminase_GpA(11-294)<br><br>YbcX (11-283)	1.86e-17<br><br>4.23e-05	COG5525; YbcX; Phage terminase, large subunit GpA [Mobilome: prophages, transposons]. (100.0/2.20e-46)<br><br>PF05876.13; Terminase_GpA; Phage terminase large subunit (GpA) (100.0/6.50e-41)<br><br>PF03354.16; Terminase_1; Phage Terminase (99.84/1.00e-18)	terminase large subunit
79	51454	52299	+	Phage protein [*Enterococcus* phage 163]<br><br>Phage protein [*Enterococcus* phage 156]<br><br>unknown [*Listeria* phage 20422-1]	0.0e+00<br><br>1.0e-77<br><br>6.0e-28	-	-	cd10676; GNAT_1Y9W; GNAT. Acetyltransferase (GNAT) family. This family contains proteins with N-acetyltransferase functions. (97.01/4.60e-02)<br><br>PF12568.9; PanZ; Acetyltransferase (GNAT) domain, PanZ(96.29/6.10e-02)<br><br>d2g3aa1; d.108.1.1 (A:1-137) Probable acetyltransferase Atu2258 {*Agrobacterium tumefaciens* [TaxId: 358]} (96.7/7.30e-02)	putative N-acetyltransferase
80	52296	53123	+	Phage protein [*Enterococcus* phage 163]	0.0e+00<br><br>3.0e-66	-	-	('-', '-')	hp

				Phage protein [*Enterococcus* phage 156]					
81	53124	53480	+	hp	hp	-	-	('-', '-')	hp
82	53585	54853	+	Phage N-acetylmuramoyl-L-alanine amidase (EC [*Enterococcus* phage 163]<br><br>putative N-acetylmuramoyl-L-alanine amidase [*Enterococcus* phage EFP01]<br><br>N-acetylmuramoyl-L-alanine amidase [*Enterococcus* phage EFDG1]	0.0e+00<br><br>0.0e+00<br><br>0.0e+00	Amidase_2 (24-155)<br><br>AmpD (1-254)	4.64e-24<br><br>7.28e-08	d1yb0a1; d.118.1.1 (A:1-157) N-acetylmuramoyl-L-alanine amidase PlyG {*Anthrax bacillus* (*Bacillus anthracis*) [TaxId: 1392]} (99.63/1.90e-14)<br><br>d1lbaa_; d.118.1.1 (A:) Bacteriophage T7 lysozyme (Zn amidase) {Bacteriophage T7 [TaxId: 10760]} (99.55/1.80e-13)<br><br>d2cb3a1; d.118.1.1 (A:174-344) Peptidoglycan-recognition protein-LE {Fruit fly (*Drosophila melanogaster*) [TaxId: 7227]} (99.53/3.10e-13)	N-acetylmuramoyl-L-alanine amidase
83	55011	55637	+	peptidoglycan-binding LysM [*Enterococcus* phage EFP01]<br><br>Aggregation promoting factor [*Enterococcus* phage 163]<br><br>aggregation promoting factor [*Enterococcus* phage EFDG1]	2.0e-119<br><br>8.0e-116<br><br>2.0e+115	LysM (35-81)<br><br>spore_safA (39-82)<br><br>PRK13914 (29-149)	1.62e-07<br><br>3.23e-07<br><br>6.91e-04	PF06737.15; Transglycosylas; Transglycosylase-like domain (98.4/6.60e-07)<br><br>d1gd6a_; d.2.1.2 (A:) Lysozyme {Silkworm (*Bombyx mori*) [TaxId: 7091]} (97.04/3.90e-03)<br><br>d1qqya_; d.2.1.2 (A:) Lysozyme {Dog (Canis familiaris), milk [TaxId: 9615]} (96.0/4.60e-02)	LysM domain containing peptidoglycan-binding protein

								KOG2850 Predicted peptidoglycan-binding protein, contains LysM domain [General function prediction only] (90.19/1.7)	
84	55741	56685	+	N-acetylmuramoyl-L-alanine amidase [*Enterococcus* phage EFDG1]<br><br>N-acetylmuramoyl-L-alanine amidase [*Enterococcus* phage PEf771]<br><br>n-acetylmuramoyl-L-alanine amidase [*Enterococcus* phage EfV12-phi1]	0.0e+00<br><br>0.0e+00<br><br>0.0e+00	Amidase_5(5-153)<br><br>SH3_5(244-302)	6.92e-38<br><br>5.25e-06	COG0791; Spr; Cell wall-associated hydrolase, NlpC family [Cell wall/membrane/envelope biogenesis]. (98.58/2.40e-06)<br><br>COG3234; *yfa*T; Uncharacterized conserved protein YfaT, DUF1175 family [Function unknown]. (98.24/2.80e-05)<br><br>PF05382.14; Amidase_5; Bacteriophage peptidoglycan hydrolase (98.28/2.8e-04)<br>PF08460.11; SH3_5; Bacterial SH3 domain (97.17/7.7e-03)	N-acetylmuramoyl-L-alanine amidase
85	56815	57159	+	hp	hp	-	-	PF06096.12; Baculo_8kDa; Baculoviridae 8.2 KDa protein (61.23/3.20e+00)	hp
86	57176	58864	+	Phage protein [*Enterococcus* phage 163]	0.0e+00<br><br>0.0e+00	Phage_portal (245-453)<br><br>portal_HK97 (121-459)	1.10e-17<br><br>1.13e-07	COG4695; BeeE; Phage portal protein BeeE [Mobilome: prophages, transposons]. (100.0/1.10e-30)	portal protein

				portal protein [*Enterococcus* phage EFDG1]<br><br>portal protein [*Enterococcus* phage PEf771]	0.0e+00	BeeE (245-467)	3.53e-06	PF04860.13; Phage_portal; Phage portal protein (99.97/5.90e-29)<br><br>COG5518; COG5518; Bacteriophage capsid portal protein [Mobilome: prophages, transposons]. (99.97/2.90e-28)	
87	58964	59752	+	prohead protease [*Enterococcus* phage EFDG1]<br><br>prohead protease [*Enterococcus* phage ECP3]<br><br>Phage protein [*Enterococcus* phage 156]	0.0e+00<br><br>1.0e+140<br><br>5.0e+140	-	-	COG3740; COG3740; Phage head maturation protease [Mobilome: prophages, transposons]. (99.88/8.10e-21)<br><br>PF04586.18; Peptidase_S78; Caudovirus prohead serine protease (99.79/3.30e-18)<br><br>PF14550.7; Peptidase_S78_2; Putative phage serine protease XkdF(98.9/1.60e-08)	prohead protease
88	59752	60771	+	hp	hp	-	-	('-', '-')	hp

89	60894	62315	+	major capsid protein [*Enterococcus* phage EfV12-phi1]<br><br>major capsid protein [*Enterococcus* phage vB_OCPT_Ben]<br><br>major capsid protein [*Enterococcus* phage EFDG1]	0.0e+00<br><br>0.0e+00<br><br>0.0e+00	-	-	PF17236.3; DUF5309; Family of unknown function (DUF5309) (99.89/1.10e-21)<br><br>d2fsya1; d.183.1.1 (A:104-383) Major capsid protein gp5 {Bacteriophage HK97 [TaxId: 37554]} (98.57/1.40e-06)<br><br>COG4653; COG4653; Predicted phage phi-C31 gp36 major capsid-like protein [Mobilome: prophages, transposons]. (97.68/1.30e-03)	major capsid protein
90	62413	62664	+	putative lipoprotein [*Listeria* phage LMSP-25]<br><br>putative lipoprotein [*Listeria* phage LMTA-34]<br><br>gp87 [*Listeria* virus A511	5.0e-07<br><br>1.0e-06<br><br>2.0e-06	-	-	('-', '-')	hp

91	62676	63572	+	Phage protein [*Enterococcus* phage 163]<br><br>structural protein [*Enterococcus* phage PBEF129]<br><br>gp88 [*Listeria* virus A511]	0.0e+00<br><br>0.0e+00<br><br>4.0e-135	-	-	PF11436.9; DUF3199; Protein of unknown function (DUF3199) (97.67/6.80e-04)<br><br>PF05135.14; Phage_connect_1; Phage gp6-like head-tail connector protein (97.23/9.50e-04)<br><br>d1xn8a_; a.229.1.1 (A:)hp YqbG {*Bacillus subtilis* [TaxId: 1423]} (97.58/1.10e-03)	head completion protein
92	63586	64458	+	Phage protein [*Enterococcus* phage 163]<br><br>capsid protein [*Enterococcus* phage PBEF129]<br><br>gp89 [*Listeria* virus A511]	0.0e+00<br><br>6.0e-138<br><br>3.0e-75	-	-	PF17302.3; DUF5351; Family of unknown function (DUF5351) (85.4/7.10e-01)	hp
93	64451	65074	+	gp90 [*Listeria* virus A511]<br><br>ORF5 [*Listeria* virus A511]<br><br>ORF055 [*Staphylococcus* virus Twort]	5.0e-70<br><br>5.0e-59<br><br>2.0e-46	-	-	('-', '-')	hp

94	65078	65923	+	baseplate hub assembly protein [*Enterococcus* phage PEf771]<br><br>Phage protein [*Enterococcus* phage 156]<br><br>gp91 [*Listeria* virus A511]	0.0e+00<br><br>1.0e-146<br><br>8.0e-76	-	-	('-', '-')	hp
95	65923	66162	+	Phage protein [*Enterococcus* phage 163]	3.0e-46	-	-	('-', '-')	hp
96	66166	67875	+	major tail sheath protein [*Enterococcus* phage EFP01]<br><br>Phage tail sheath [*Enterococcus* phage 163]<br><br>Phage major tail sheath [*Enterococcus* phage 156]	0.0e+00<br><br>0.0e+00<br><br>0.0e+00	-	-	COG3497; COG3497; Phage tail sheath protein FI [Mobilome: prophages, transposons]. (99.95/5.80e-25)<br><br>COG4386; COG4386; Mu-like prophage tail sheath protein gpL [Mobilome: prophages, transposons]. (99.96/5.90e-25)<br><br>PF04984.15; Phage_sheath_1; Phage tail sheath protein subtilisin-like domain (99.05/9.5e-9)	tail sheath protein
97	67941	68363	+	tail tube protein [*Enterococcus* phage EFDG1]<br><br>tail tube protein [*Enterococcus* phage EF24C]	1.0e-91<br><br>1.0e-89	-	-	PF10772.10; DUF2597; Protein of unknown function (DUF2597) (99.83/1.40e-19)<br><br>d2guja1; b.106.1.3 (A:5-143) Phage-like element PBSX	tail tube protein

				tail tube protein [*Enterococcus* phage ECP3]	1.0e-89			protein XkdM {*Bacillus subtilis* [TaxId: 1423]} (99.65/2.40e-14)<br><br>PF09393.11; DUF2001; Phage tail tube protein (99.45/1.40e-11) PF06199.12; Phage_tail_2; Phage tail tube protein (96.76/0.11)	
98	68454	69566	+	Phage protein [*Enterococcus* phage 156]<br><br>ORF018 [*Staphylococcus* virus Twort]<br><br>unknown [*Staphylococcus* virus Twort]	0.0e+00<br><br>1.0e-36<br><br>7.0e-34	-	-	d1vsra_; c.52.1.15 (A:) Very short patch repair (VSR) endonuclease {*Escherichia coli* [TaxId: 562]} (98.51/1.40e-06)<br><br>PF14311.7; DUF4379; Probable Zinc-ribbon domain (97.98/4.60e-06)<br><br>COG3727; Vsr; G:T-mismatch repair DNA endonuclease, very short patch repair protein [Replication, recombination and repair]. (98.29/8.70e-06)	VSR endonuclease
99	69823	70296	+	hp EFP01_076 [*Enterococcus* phage EFP01]<br><br>tail tape measure protein [*Enterococcus* phage EFDG1]	7.0e-105<br><br>2.0e-101<br><br>1.0e-31	-	-	PF10109.10; Phage_TAC_7; Phage tail assembly chaperone proteins, E, or 41 or 14 (39.22/130)	putative tail assembly chaperone protein

				tail assembly chaperone [*Bacillus* phage Chotacabras]					
100	70379	70945	+	Phage protein [*Enterococcus* phage 163]<br><br>putative RNA polymerase [*Enterococcus* phage EFP01]<br><br>tail assembly chaperone protein [*Bacillus* phage Bobb]	5.0e-121<br><br>3.0e-119<br><br>3.0e-14	-	-	('-', '-')	hp
101	70995	74642	+	tail length tape-measure protein [*Enterococcus* phage vB_OCPT_Ben]<br><br>tail length tape-measure protein [*Enterococcus* phage EFDG1]<br><br>tail length tape-measure protein [*Enterococcus* phage EfV12-phi1]	0.0e+00<br><br>0.0e+00<br><br>0.0e+00	LT_TF-like (988-1117)<br><br>SLT (989-1179)<br><br>Smc (18-281)	9.91e-30<br><br>2.36e-11<br><br>6.23e-09	COG5283; COG5283; Phage-related tail protein [Mobilome: prophages, transposons]. (99.65/2.40e-10)<br><br>COG5280; YqbO; Phage-related minor tail protein [Mobilome: prophages, transposons]. (99.19/5.00e-06)<br><br>COG3941 HI1514; Phage tail tape-measure protein, controls tail length [Mobilome: prophages, transposons]. (98.82/3e-04)	lytic transglycosylase-like domain containing tail tape measure protein
102	74692	77211	+	repressor protein C2 [*Enterococcus* phage EFP01]	0.0e+00	NLPC_P60 (706-835)	1.52e-13	COG0791; Spr; Cell wall-associated hydrolase, NlpC family [Cell	peptidoglycan hydrolase domain containing

				Secretory antigen SsaA-like protein [*Enterococcus* phage 163]<br><br>putative tail lysin [*Enterococcus* phage EfsSzw-1]	0.0e+00<br><br>0.0e+00	PRK13914 (705-807)	3.15e-05	wall/membrane/envelope biogenesis]. (99.21/1.00e-10)<br><br>COG4379 COG4379; Mu-like prophage tail protein gpP [Mobilome: prophages, transposons]. (98.35/4.3e-04)<br><br>PF05382.14; Amidase_5; Bacteriophage peptidoglycan hydrolase (97.61/1e-03)<br><br>PF14594.7; Sipho_Gp37; Siphovirus ReqiPepy6 Gp37-like protein (97.51/7.7e-02)	baseplate hub protein
103	77311	84240	+	Glycerophosphoryl diester phosphodiesterase (EC, phage variant [*Enterococcus* phage 163]<br><br>glycerophosphoryl diester phosphodiesterase [*Enterococcus* phage EFDG1]<br><br>structural component of the tail fiber [*Enterococcus* phage PEf771]<br><br>putative tail fiber [*Enterococcus* phage EFP01]	0.0e+00<br><br>0.0e+00<br><br>0.0e+00<br><br>0.0e+00	-	-	d1k28a1 b.40.8.1 (A:6-129) Tail-associated lysozyme gp5, N-terminal domain {Bacteriophage T4 [TaxId: 10665]} (88.38/1.1)<br><br>PF18352.2; Gp138_N; Phage protein Gp138 N-terminal domain (87.26/0.8)<br><br>COG4384 gp45; Mu-like prophage protein gp45 [Mobilome: prophages, transposons]. (81.16/5.3)<br><br>PF04717.13; Phage_base_V; Type VI secretion system/phage-	putative central tail fiber

								baseplate injector OB domain (58.03/50)	
104	84333	86744	+	Phage capsid and scaffold [*Enterococcus* phage 163]<br><br>capsid and scaffold protein [*Enterococcus* phage vB_OCPT_Ben]<br><br>capsid and scaffold protein [*Enterococcus* phage EFDG1]<br><br>minor tail protein [*Bacillus* phage 035JT001]<br><br>tail fiber protein [*Bacillus* phage Beyonphe]	0.0e+00<br><br>0.0e+00<br><br>0.0e+00<br><br>1.0e-86<br><br>1.0e-62	DUF859 (13-696)	1.45e-27	PF05895.13; DUF859; Siphovirus protein of unknown function (DUF859) (100.0/1.30e-67)	DUF859 containing protein
105	86741	87484	+	Phage protein [*Enterococcus* phage 156]	6.0e-33	-	-	PF14301.7; DUF4376; Domain of unknown function (DUF4376) (99.15/2.70e-09)	hp
106	87505	87651	+	Phage protein [*Enterococcus* phage 163]	5.0e-27	-	-	PF12512.9; DUF3717; Protein of unknown function (DUF3717) (92.66/5.30e-01)	hp
107	87806	88507	+	Phage protein [*Enterococcus* phage 163]<br><br>gp100 [*Listeria* virus A511]<br>ORF041 [*Staphylococcus* virus Twort]	7.0e-173<br><br>1.0e-79<br><br>3.0e-46	-	-	COG3499; COG3499; Phage protein U [Mobilome: prophages, transposons]. (97.11/4.20e-03)<br><br>PF06995.12; Phage_P2_GpU; Phage P2 GpU (94.64/0.89)	tail tube terminator protein

108	88507	89043	+	Phage protein [*Enterococcus* phage 156]<br><br>gp101 [*Listeria* virus A511]<br><br>structural protein [*Staphylococcus* phage pSco-10]	4.0e-110<br><br>2.0e-52<br><br>5.0e-30	-	-	('-', '-')	hp
109	89030	89734	+	Phage baseplate assembly protein [*Enterococcus* phage 163]<br><br>putative baseplate [*Enterococcus* phage EfsSzw-1]<br><br>putative baseplate [*Enterococcus* phage EFP01]<br><br>baseplate wedge subunit [*Staphylococcus* phage Stab23]	6.0e-174<br><br>1.0e-172<br><br>4.0e-172<br><br>3.0e-71	-	-	PF10934.9; DUF2634; Protein of unknown function (DUF2634) (99.61/2.20e-14)<br><br>COG3628; COG3628; Phage baseplate assembly protein W [Mobilome: prophages, transposons]. (99.48/3.70e-12)<br><br>d2ia7a1; d.373.1.1 (A:23-133) Uncharacterized protein GSU0986 {*Geobacter sulfurreducens* [TaxId: 35554]} (99.46/4.30e-12)<br><br>PF11246.9; Phage_gp53; Base plate wedge protein 53 (26.31/190)	baseplate assembly protein
110	89749	90801	+	Phage baseplate [*Enterococcus* phage 163]	0.0e+00<br><br>0.0e+00	Baseplate_J (55-311)<br><br>JayE(118-350)	2.41e-16<br><br>5.33e-05	COG3299; JayE; Uncharacterized phage protein gp47/JayE [Mobilome: prophages, transposons]. (100.0/4.70e-34)	baseplate assembly protein

				putative baseplate J protein [*Enterococcus* phage EFP01]<br><br>baseplate protein [*Enterococcus* phage EfV12-phi1]	0.0e+00			COG3948; COG3948; Phage-related baseplate assembly protein [Mobilome: prophages, transposons]. (99.97/5.50e-30)<br><br>PF04865.15; Baseplate_J; Baseplate J-like protein (99.97/1.00e-28)	
111	90813	95441	+	Phage protein [*Enterococcus* phage 163]<br><br>Phage protein [*Enterococcus* phage 156]<br><br>gp104 [*Listeria* virus A511]<br><br>putative tail fiber protein complex [*Staphylococcus* phage KSAP7]<br><br>tail morphogenetic protein [*Staphylococcus* phage pSco-10]<br><br>minor tail protein [*Staphylococcus* phage Metroid]	0.0e+00<br><br>2.0e-154<br><br>2.0e-81<br><br>5.0e-71<br><br>9.0e-71<br><br>1.0e-70	Big_2 (1130-1202)<br><br>YjdB (1130-1206)	2.42e-11<br><br>2.28e-09	PF11041.9; DUF2612; Protein of unknown function (DUF2612) (99.14/4.70e-10)<br><br>PF10076.10; DUF2313; Uncharacterised protein conserved in bacteria (DUF2313) (98.99/5.90e-09)<br><br>PF09684.11; Tail_P2_I; Phage tail protein (Tail_P2_I) (97.74/1.60e-04)<br><br>COG4385 gpI; Bacteriophage P2-related tail formation protein [Mobilome: prophages, transposons]. (97.56/7.7e-04)<br><br>COG3299 JayE; Uncharacterized phage protein gp47/JayE [Mobilome: prophages, transposons]. (96.76/8.6e-03)	baseplate assembly protein

								5IW9_B; Baseplate wedge protein gp25; contractile sheath, baseplate, wedge, sheath; HET: MSE; 2.47A {*Enterobacteria* phage T4}; (99.57/1.40e-13)	
112	95555	96100	+	Phage protein [*Enterococcus* phage 163]<br><br>structural protein [*Enterococcus* phage PEf771]<br><br>structural protein [*Enterococcus* phage EFP01]	7.0e-134<br><br>2.0e-132<br><br>4.0e-131	-	-	PF09215.11; Phage-Gp8; Bacteriophage T4, Gp8(99.86/7.10e-21)<br><br>COG5301; COG5301; Phage-related tail fibre protein [Mobilome: prophages, transposons]. (98.27/3.50e-05)<br><br>d1n7za_; b.127.1.1 (A:) Baseplate structural protein gp8 {Bacteriophage T4 [TaxId: 10665]} (97.86/1.20e-03)<br><br>6HHK_A; Gp105; bacteriophage baseplate protein, VIRAL PROTEIN; HET: MSE; 2.38A {*Listeria* phage A511}; (94.97/2.20e-02)	baseplate assembly protein
113	96117	97163	+	Phage protein [*Enterococcus* phage 156]<br><br>group I intron protein [*Bacillus* phage Bobb]	1.0e-34<br><br>6.0e-17	-	-	PF01710.17; HTH_Tnp_IS630; Transposase (98.37/4.90e-06)<br><br>d1vsra_; c.52.1.15 (A:) Very short patch repair (VSR) endonuclease {*Escherichia coli* [TaxId: 562]} (98.39/5.60e-06)	VSR endonuclease

				DNA-binding protein [*Bacillus* phage Moonbeam]<br><br>homing endonuclease [*Staphylococcus* phage pSco-10]	6.0e-14<br><br>9.0e-11			PF04936.13; DUF658; Protein of unknown function (DUF658) (97.82/1.90e-05)<br><br>COG2852 YcjD; Very-short-patch-repair endonuclease [Replication, recombination and repair]. (97.73/4.9e-05)	
114	97166	100624	+	virulence-associated protein [*Enterococcus* phage vB_OCPT_Ben]<br><br>virulence-associated protein [*Enterococcus* phage EfV12-phi1]<br><br>putative adsorption associated tail protein [*Enterococcus* phage EFP01]	0.0e+00<br><br>0.0e+00<br><br>0.0e+00	DUF4815 (13-613)	0e+00	PF16075.6; DUF4815; Domain of unknown function (DUF4815) (100.0/4.80e-110)	putative adsorption associated tail protein
115	100705	101667	+	HNH endonuclease [*Staphylococcus* phage vB_SepS_SEP9]<br><br>putative HNH endonuclease [Acinetobacter phage YMC-13-01-C62]	1.0e-49<br><br>2.0e-41<br><br>9.0e-40	-	-	PF07768.12; PVL_ORF50; PVL ORF-50-like family (98.15/6.70e-06)<br><br>PF07768.12; PVL_ORF50; PVL ORF-50-like family (97.52/3.10e-04)<br><br>PF04936.13; DUF658; Protein of unknown function (DUF658) (95.84/9.50e-02)	PVL ORF-50-like family protein

				putative HNH homing endonuclease [*Bacillus* phage Izhevsk]					
116	101745	101951	+	gp107 [*Listeria* virus A511]<br><br>gp17.1 [*Bacillus* virus SPO1]	1.0e-06<br><br>5.0e-03	-	-	KOG4098; Molecular chaperone Prefoldin, subunit 2 [Posttranslational modification, protein turnover, chaperones] (94.07/6.50e-01)	hp
117	102144	105191	+	putative helicase [*Enterococcus* phage EFP01]<br><br>DNA helicase [*Enterococcus* phage PBEF129]<br><br>DNA helicase, phage-associated [*Enterococcus* phage 156]	0.0e+00<br><br>0.0e+00<br><br>0.0e+00	uvsW (745-1012)<br><br>Intein_splicing (179-576)<br><br>Hop (210-576)<br><br>Helicase_C 872-967	6.66e-31<br><br>1.40e-22<br><br>3.25e-20<br><br>9.18e-13	KOG0339; ATP-dependent RNA helicase [RNA processing and modification] (99.93/8.40e-26)<br><br>KOG0338; ATP-dependent RNA helicase [RNA processing and modification] (99.93/1.10e-25)<br><br>KOG0346; RNA helicase [RNA processing and modification] (99.94/1.70e-25)<br><br>2OCA_A; ATP-dependent DNA helicase uvsW (E.C.3.6.1.8); ATP-dependent helicase, T4-bacteriophage, Recombination, HYDROLASE; 2.7A {*Enterobacteria* phage T4}(99.98/2.00e-30)	uvsW-like ATP-dependent DNA helicase

118	105207	106850	+	putative transcriptional regulator [*Enterococcus* phage EFP01]<br><br>putative transcriptional regulator [*Enterococcus* phage EfsSzw-1]<br><br>putative transcriptional regulator [*Enterococcus* phage phiEF17H]	0.0e+00<br><br>0.0e+00<br><br>0.0e+00	HTH_36 (27-71)	5.32e-04	d2gaua1; a.4.5.4 (A:152-232) Transcriptional regulator PG0396, C-terminal domain {*Porphyromonas gingivalis* [TaxId: 837]} (97.81/1.50e-04)<br><br>d1ub9a_; a.4.5.28 (A:)hp PH1061 {*Pyrococcus horikoshii* [TaxId: 53953]} (97.56/1.90e-04)<br><br>d3e5ua1; a.4.5.4 (A:148-227) Chlorophenol reduction protein CprK {*Desulfitobacterium hafniense* [TaxId: 49338]} (97.68/2.20e-04)	putative transcriptional regulator
119	106864	108336	+	DNA helicase [*Enterococcus* phage EfV12-phi1]<br><br>DNA helicase [*Enterococcus* phage vB_OCPT_Ben]<br><br>helicase/primase [*Enterococcus* phage PEf771]<br><br>helicase DnaB-like [*Enterococcus* phage EFDG1]	0.0e+00<br><br>0.0e+00<br><br>0.0e+00<br><br>0.0e+00	41 (70-480)<br><br>phage_DnaB (157-380)<br><br>DnaB_C (190-380)	5.11e-16<br><br>5.46e-12<br><br>1.83e-10	COG0305; DnaB; Replicative DNA helicase [Replication, recombination and repair]. (100.0/9.30e-32)<br><br>d1cr1a_; c.37.1.11 (A:) Gene 4 protein (g4p, DNA primase), helicase domain {Bacteriophage T7 [TaxId: 10760]} (99.92/3.00e-22)<br><br>PF03796.16; DnaB_C; DnaB-like helicase C terminal domain (99.89/2.80e-20)	DnaB-like replicative helicase

120	108336	109376	+	putative exonuclease [*Enterococcus* phage EFP01]<br><br>recombination exonuclease [*Enterococcus* phage EfV12-phi1]<br><br>Phage recombination exonuclease [*Enterococcus* phage 163]	0.0e+00<br><br>0.0e+00<br><br>0.0e+00	MPP_Mre11_N (8-223)<br><br>SbcD (1-313)<br><br>sbcd (1-231)	7.93e-24<br><br>5.65e-21<br><br>3.30e-14	COG0420; SbcD; DNA repair exonuclease SbcCD nuclease subunit [Replication, recombination and repair]. (99.95/9.20e-25)<br><br>d3dsda_; d.159.1.4 (A:) Mre11 {*Pyrococcus furiosus* [TaxId: 2261]} (99.94/2.70e-24)<br><br>KOG2310; DNA repair exonuclease MRE11 [Replication, recombination and repair] (99.9/1.10e-21)	DNA repair exonuclease SbcCD-like nuclease subunit
121	109495	111387	+	Phage recombination related exonuclease (EC [*Enterococcus* phage 163]<br><br>putative exonuclease [*Enterococcus* phage EFP01]<br><br>recombination endonuclease [*Enterococcus* phage EFDG1]	0.0e+00<br><br>0.0e+00<br><br>0.0e+00	46 (1-619)<br><br>SbcC (3-424)<br><br>sbcc (42-459)	2.92e-45<br><br>4.02e-25<br><br>7.53e-21	COG0419; SbcC; DNA repair exonuclease SbcCD ATPase subunit [Replication, recombination and repair]. (100.0/1.20e-29)<br><br>COG5293; YdbB; Uncharacterized protein YdbD, contains DUF2326 domain [Function unknown]. (100.0/4.30e-28)<br><br>KOG0962; DNA repair protein RAD50, ABC-type ATPase/SMC superfamily [Replication, recombination and repair] (100.0/4.40e-28)	DNA repair exonuclease SbcCD-like ATPase subunit

122	111401	112060	+	Phage protein [*Enterococcus* phage 163]<br><br>putative repressor [*Enterococcus* phage EFP01]<br><br>putative repressor [*Enterococcus* phage PBEF129]	1.0e-154<br><br>5.0e-151<br><br>4.0e-81	-	-	('-', '-')	hp
123	112064	113119	+	Phage DNA primase/helicase [*Enterococcus* phage 163]<br><br>putative primase [*Enterococcus* phage EfsSzw-1]<br><br>putative primase [*Enterococcus* phage EFP01]	0.0e+00<br><br>0.0e+00<br><br>0.0e+00	dnaG (50-325)<br><br>dnaG (46-293)<br><br>DnaG (50-328)	3.42e-17<br><br>4.39e-14<br><br>1.75e-12	COG0358; DnaG; DNA primase (bacterial type) [Replication, recombination and repair]. (100.0/8.90e-34)<br><br>d1dd9a_; e.13.1.1 (A:) DNA primase DnaG catalytic core {*Escherichia coli* [TaxId: 562]} (99.93/3.00e-24)<br><br>COG4643; COG4643; Uncharacterized domain associated with phage/plasmid primase [Mobilome: prophages, transposons]. (99.9/1.30e-22)<br><br>2AU3_A; DNA primase (E.C.2.7.7.-); Zinc Ribbon, TOPRIM, RNA POLYMERASE; 2.0A {*Aquifex aeolicus*}(99.97/3.10e-29)	DNA primase

124	113129	114010	+	Deoxyuridine 5'-triphosphate nucleotidohydrolase (EC [*Enterococcus* phage 163]<br><br>deoxyuridine 5'-triphosphate nucleotidohydrolase [*Enterococcus* phage vB_OCPT_Ben]<br><br>deoxyuridine 5'-triphosphate nucleotidohydrolase [*Enterococcus* phage EFDG1]	0.0e+00<br><br>0.0e+00<br><br>0.0e+00	Dut (83-292)<br><br>trimeric_dUTPase (105-176)	2.68e-14<br><br>1.11e-06	COG0756; Dut; dUTPase [Nucleotide transport and metabolism, Defense mechanisms]. (99.93/1.10e-24)<br><br>KOG3370; dUTPase [Nucleotide transport and metabolism] (99.93/1.50e-24)<br><br>d1sixa1; b.85.4.1 (A:1-144) Deoxyuridine 5'-triphosphate nucleotidohydrolase (dUTPase) {*Mycobacterium tuberculosis*, rv2697c [TaxId: 1773]} (99.93/3.70e-24)	dUTPase
125	114010	114246	+	putative dUTPase [*Enterococcus* phage vB_EfaH_EF1TV]<br><br>putative dUTPase [*Enterococcus* phage EF24C]<br><br>putative dUTPase [*Enterococcus* phage ECP3]	1.0e-07<br><br>5.0e-07<br><br>9.0e-07	-	-	('-', '-')	hp
126	114250	114561	+	Phage protein [*Enterococcus* phage 156]	6.0e-04	-	-	('-', '-')	hp
127	114548	114859	+	putative phosphotransferase/anion		-	-	d1nh2d1; a.32.1.1 (D:5-54) Small chain TOA2, N-terminal	hp

				transport protein [*Enterococcus* phage EFP01]<br><br>putative phosphotransferase/anion transport protein [*Enterococcus* phage EF24C]<br><br>putative phosphotransferase/anion transport protein [*Enterococcus* phage vB_EfaH_EF1TV]	4.0e-67<br><br>7.0e-12<br><br>1.0e-11			domain {Baker's yeast (*Saccharomyces cerevisiae*) [TaxId: 4932]} (84.88/3.00e+00)	
128	11485<br>2	11522<br>0	+	Phage protein (ACLAME 1549) [*Enterococcus* phage 163]<br><br>gp121 [*Listeria* virus A511]	2.0e-82<br><br>2.0e-13	PHA02277 (11-117)	6.24e-05	d2elca1; a.46.2.1 (A:1-65) Anthranilate phosphoribosyltransferase (TrpD) {*Thermus thermophilus* [TaxId: 274]} (96.46/3.30e-02)<br><br>d4eada1; a.46.2.1 (A:2-70) Thymidine phosphorylase {*Escherichia coli* [TaxId: 562]} (96.43/3.60e-02)<br><br>d1k8ea1; a.46.2.1 (A:1-70) Anthranilate phosphoribosyltransferase (TrpD) {*Sulfolobus solfataricus* [TaxId: 2287]} (96.33/4.10e-02)	hp

129	115228	115800	+	hp	hp	-	-	('-', '-')	hp
130	115793	116770	+	VSR homing endonuclease [*Staphylococcus* phage Quidividi]<br><br>homing endonuclease [*Bacillus* virus PBS1]<br><br>group I intron-associated VSR homing endonuclease [*Staphylococcus* phage Terranova]	8.0e-47<br><br>2.0e-45<br><br>8.0e-44	DUF2726 (238-310)	1.13e-05	d1vsra_; c.52.1.15 (A:) Very short patch repair (VSR) endonuclease {*Escherichia coli* [TaxId: 562]} (98.8/4.70e-08)<br><br>COG3727; Vsr; G:T-mismatch repair DNA endonuclease, very short patch repair protein [Replication, recombination and repair]. (98.67/4.20e-07)<br><br>COG2852; YcjD; Very-short-patch-repair endonuclease [Replication, recombination and repair]. (98.35/5.10e-06)	VSR endonuclease
131	116861	117445	+	putative resolvase [*Enterococcus* phage EFP01]<br><br>Phage protein [*Enterococcus* phage 163]<br><br>putative resolvase [*Enterococcus* phage ECP3]	5.0e-143<br><br>2.0e-138<br><br>3.0e-100	-	-	d1ob8a_; c.52.1.18 (A:) Holliday-junction resolvase SSO1176 {*Sulfolobus solfataricus* [TaxId: 2287]} (98.36/3.50e-06)<br><br>COG1591; COG1591; Holliday junction resolvase, archaeal type [Replication, recombination and repair]. (97.81/1.80e-04)<br><br>d1gefa_; c.52.1.18 (A:) Archaeal Holliday junction resolvase Hjc {*Pyrococcus furiosus* [TaxId: 2261]} (97.62/8.10e-04)	Holliday-junction resolvase

132	117445	117810	+	hp	hp	-	-	('-', '-')	hp
133	117833	117970	+	hp	hp	-	-	('-', '-')	hp
134	117967	118389	+	Phage protein [*Enterococcus* phage 156]<br><br>ORF050 [*Staphylococcus* virus 3a]<br><br>ORF060 [*Staphylococcus* virus 96]	6.0e-32<br><br>4.0e-15<br><br>4.0e-15	DUF3310 (7-66)	2.83e-23	PF11753.9; DUF3310; Protein of unknwon function (DUF3310) (99.78/5.10e-19)	DUF3310 containing protein
135	118495	119289	+	Phage protein [*Enterococcus* phage 156]<br>gp135 [*Listeria* virus A511]<br>synaptonemal complex 1 (SCP-1) domain containing protein [*Listeria* phage LMTA-148]	4.0e-148<br><br>1.0e-89<br><br>2.0e-89	-	-	cd17835; Radical_SAM_spore_photoproduct_lyase; spore photoproduct lyase. Radical SAM superfamily. Enzymes of this family generate radicals by combining a 4Fe-4S cluster and S-adenosylmethionine (SAM) in close proximity. (91.55/6.10e+00)	hp
136	119282	119593	+	integration host factor [*Enterococcus* phage EFDG1]<br><br>putative integration host factor [*Enterococcus* phage EfsSzw-1]	3.0e-71<br><br>1.0e-70<br><br>4.0e-59	Bac_DNA_binding(8-95)<br><br>IHF (9-93)	9.00e-14<br><br>1.82e-06	d1mula_; a.55.1.1 (A:) HU protein {*Escherichia coli* [TaxId: 562]} (99.91/5.40e-23)<br><br>d1p71a_; a.55.1.1 (A:) HU protein {Anabaena sp. [TaxId: 1167]} (99.91/1.20e-22)	integration host factor

				putative integration host factor [*Enterococcus* phage EF24C]				d1owfa_ a.55.1.1 (A:) Integration host factor alpha subunit (IHFA) {*Escherichia coli* [TaxId: 562]} (99.91/3.7e-22)	
137	119674	120516	+	DNA polymerase I [*Enterococcus* phage EfV12-phi1]<br><br>putative DNA polymerase [*Enterococcus* phage EfsSzw-1]<br><br>DNA polymerase I [*Enterococcus* phage PEf771]	0.0e+00<br><br>0.0e+00<br><br>0.0e+00	DNA_polA_I_Ecoli_like_exo (207-280)<br><br>PRK05755 (167-280)<br><br>UDG-F4_TTUDGA_SPO1dp_like (97-169)	1.31e-09<br><br>5.32e-08<br><br>2.61e-04	d1vk2a_; c.18.1.2 (A:) Thermophilic uracil-DNA glycosylase {*Thermotoga maritima* [TaxId: 2336]} (99.86/2.70e-20)<br><br>d1ui0a_; c.18.1.2 (A:) Thermophilic uracil-DNA glycosylase {*Thermus thermophilus* [TaxId: 274]} (99.84/7.30e-20)<br><br>COG1573; Udg4; Uracil-DNA glycosylase [Replication, recombination and repair]. (99.83/1.90e-19)<br><br>COG0749 PolA; DNA polymerase I - 3'-5' exonuclease and polymerase domains [Replication, recombination and repair]. (97.07/0 8e-03)	3'-5' exonuclease and polymerase domains containing uracil DNA glycosylase family 4
138	120687	121163	+	gp86 [*Bacillus* phage W.Ph.]	5.0e-39	-	-	d1e7la2; d.4.1.5 (A:1-103) Recombination endonuclease VII, N-terminal domain	recombination endonuclease

				homing endonuclease [*Bacillus* phage Moonbeam]<br><br>baseplate wedge protein gp25 [*Cronobacter* phage vB_CsaM_GAP31]	2.0e-28<br><br>2.0e-18			{Bacteriophage T4 [TaxId: 10665]} (98.26/1.70e-06)<br><br>d4ogca2; d.4.1.8 (A:513-673) CRISPR-associated endonuclease Cas9/Csn1, HNH domain {*Actinomyces naeslundii* [TaxId: 1115803]} (98.05/4.30e-06)<br><br>PF09665.11; RE_Alw26IDE; Type II restriction endonuclease (RE_Alw26IDE) (97.98/4.30e-06)<br><br>PF05766.13; NinG; Bacteriophage Lambda NinG protein (97.84/1.4e-04)	
139	121242	123431	+	DNA polymerase I (EC, phage-associated [*Enterococcus* phage 163]<br><br>DNA polymerase I [*Enterococcus* phage EfV12-phi1]<br><br>DNA polymerase I [*Enterococcus* phage PEf771]	0.0e+00<br><br>0.0e+00<br><br>0.0e+00	DNA_pol_A (347-664)<br><br>DNA_pol_A_pol_I_C (261-666)<br><br>PRK05755 (137-670)	1.11e-104<br><br>2.49e-97<br><br>7.04e-83	COG0749; PolA; DNA polymerase I - 3'-5' exonuclease and polymerase domains [Replication, recombination and repair]. (100.0/2.50e-69)<br><br>d1kfsa2; e.8.1.1 (A:519-928) DNA polymerase I (Klenow fragment) {*Escherichia coli* [TaxId: 562]} (100.0/4.90e-64)<br><br>d3py8a2; e.8.1.1 (A:423-832) DNA polymerase I (Klenow	3'-5' exonuclease and polymerase domains containing DNA polymerase I

								fragment) {*Thermus aquaticus* [TaxId: 271]} (100.0/3.20e-63)	
140	123555	124085	+	gp139 [*Listeria* virus A511]<br><br>ssDNA binding protein [*Bacillus* phage Chotacabras]<br><br>ORF077 [*Staphylococcus* virus Twort]	1.0e-42<br><br>3.0e-35<br><br>6.0e-26	-	-	PF11056.9; UvsY; Recombination, repair and ssDNA binding protein UvsY (99.78/2.10e-17)	UvsY-like protein
141	124157	125473	+	Phage protein [*Enterococcus* phage 163]<br><br>Phage protein [*Enterococcus* phage 156]<br><br>gp71 [*Listeria* virus P100]	0.0e+00<br><br>3.0e-140<br><br>1.0e-45	PRK14971 (299-425)	4.82e-05	PF08804.11; gp32; gp32 DNA binding protein like (99.95/1.40e-26)	Gp32-like ssDNA binding protein
142	125554	126801	+	Phage recombinase [*Enterococcus* phage 163]<br><br>recombinase A [*Enterococcus* phage PEf771]<br><br>recombinase [*Enterococcus* phage EfV12-phi1]	0.0e+00<br><br>0.0e+00<br><br>0.0e+00	recA (38-358)<br><br>RecA (45-290)<br><br>tigrfam_recA (38-323)	1.79e-41<br><br>2.18e-35<br><br>2.46e-33	cd00983; recA; RecA is a bacterial enzyme which has roles in homologous recombination, DNA repair, and the induction of the SOS response. (100.0/3.80e-34)<br><br>PF00154.22; RecA; recA bacterial DNA recombination protein (99.96/2.40e-26)<br><br>d1u94a1; c.37.1.11 (A:6-268) RecA protein, ATPase-domain	recA protein

								{*Escherichia coli* [TaxId: 562]} (99.95/3.60e-25)	
143	126844	127218	+	Phage protein [*Enterococcus* phage 163]<br><br>Phage protein [*Enterococcus* phage 156]<br><br>gp142 [*Listeria* virus A511]	7.0e-90<br><br>6.0e-54<br><br>6.0e-19	-	-	PF06257.12; VEG; Biofilm formation stimulator VEG (94.48/5.00e-01)	hp
144	127211	127828	+	putative RNA polymerase sigma factor [*Enterococcus* phage EFP01]<br><br>putative sigma factor [*Enterococcus* phage EfsSzw-1]<br><br>putative sigma factor [*Enterococcus* phage ECP3]	3.0e-145<br><br>1.0e-141<br><br>2.0e-116	-	-	COG1595; RpoE; DNA-directed RNA polymerase specialized sigma subunit, sigma24 family [Transcription]. (99.84/3.80e-19)<br><br>COG1191; FliA; DNA-directed RNA polymerase specialized sigma subunit [Transcription]. (99.85/6.20e-19)<br><br>PF07638.12; Sigma70_ECF; ECF sigma factor (99.84/6.40e-19)	RNA polymerase sigma factor
145	127896	128174	+	holin-like protein [*Enterococcus* phage PEf771]<br><br>putative holin [*Enterococcus* phage EfsSzw-1]	3.0e-63<br><br>2.0e-62	Holin_SPP1 (13-81)	1.51e-23	PF04688.14; Holin_SPP1; SPP1 phage holin (99.9/6.70e-23)<br><br>COG5546; COG5546; Uncharacterized membrane protein [Function unknown]. (96.46/4.50e-02)	holin

				putative holin [*Enterococcus* phage EF24C]	9.0e-58			PF04531.14; Phage_holin_1; Bacteriophage holin (95.44/0.19)	
146	128223	129170	+	putative Ig-like protein [*Enterococcus* phage EFP01]<br><br>major tail protein [*Enterococcus* phage EFDG1]<br><br>major tail protein [*Enterococcus* phage EfV12-phi1]	0.0e+00<br><br>0.0e+00<br><br>0.0e+00	YjdB (195-315)<br><br>PHA02283 (13-146)<br><br>Big_2 (233-308)	3.16e-19<br><br>1.94e-18<br><br>6.30e-14	COG5492; YjdB; Uncharacterized conserved protein YjdB, contains Ig-like domain [General function prediction only]. (98.22/1.10e-03)<br><br>COG5492; YjdB; Uncharacterized conserved protein YjdB, contains Ig-like domain [General function prediction only]. (97.36/9.00e-02)	Ig-like domain containing YjdB-like protein
147	129197	129640	+	putative DNA polymerase [*Enterococcus* phage EFP01]<br><br>Phage protein [*Enterococcus* phage 163]<br><br>structural protein [*Enterococcus* phage EfsSzw-1]	4.0e-96<br><br>5.0e-96<br><br>4.0e-95	PHA02283 (1-135)	2.23e-22	('-', '-')	hp
148	129748	129984	+	Phage protein [*Enterococcus* phage 156]	5.0e-07	-	-	PF10115.10; HlyU; Transcriptional activator HlyU (86.69/6.20e+00)	putative transcriptional activator HlyU

149	129977	130246	+	hp	hp	-	-	PF07852.12; DUF1642; Protein of unknown function (DUF1642) (98.15/8.50e-06)	hp
150	130221	131159	+	Phage protein [*Enterococcus* phage 156]<br><br>gp147 [*Listeria* virus A511]<br><br>phosphodiesterase [*Bacillus* phage Kioshi]	1.0e-114<br><br>9.0e-54<br><br>3.0e-22	-	-	COG2404; NrnB; Oligoribonuclease NrnB or cAMP/cGMP phosphodiesterase, DHH superfamily [Translation, ribosomal structure and biogenesis, Signal transduction mechanisms]. (99.92/1.70e-23)<br><br>d1ir6a_; c.107.1.2 (A:) Exonuclease RecJ {*Thermus thermophilus* [TaxId: 274]} (99.92/8.50e-23)<br><br>COG1107; COG1107; Archaea-specific RecJ-like exonuclease, contains DnaJ-type Zn finger domain [Replication, recombination and repair]. (99.91/4.10e-22)	DnaJ-type Zn-binding domain containing RecJ-like exonuclease
151	131217	132500	+	Phage protein [*Enterococcus* phage 163]<br><br>metallophosphoesterase domain-containing protein [*Enterococcus* phage PEf771]	0.0e+00<br><br>0.0e+00	MPP_Mre11_N (168-391)	2.77e-05	d2a22a1; d.159.1.7 (A:4-196) MPP_DNA_pol_II_small_archeal_C; archeal DNA polymerase II, small subunit, C-terminal metallophosphatase domain. The small subunit of the archeal DNA polymerase II contains a C-terminal metallophosphatase domain. (99.57/7.90e-14)	metallophosphatase domain containing protein

				putative DNA repair exonuclease [*Enterococcus* phage EFP01]	0.0e+00			cd00144 MPP_PPP_family; phosphoprotein phosphatases of the metallophosphatase superfamily, metallophosphatase domain. (99.33/2.8e-11)<br><br>cd00840 MPP_Mre11_N; Mre11 nuclease, N-terminal metallophosphatase domain. Mre11 (also known as SbcD in *Escherichia coli*) is a subunit of the MRX protein complex. (99.51/3.1e-13)	
152	132513	132881	+	gp149 [*Listeria* virus A511]<br><br>gp81 [*Listeria* virus P100]	5.0e-07<br><br>5.0e-07	-	-	('-', '-')	hp
153	132926	133570	+	Phage protein [*Enterococcus* phage 163]<br><br>gp150 [*Listeria* virus A511]<br><br>gp118 [*Bacillus* phage W.Ph.]	1.0e-157<br><br>3.0e-23<br><br>1.0e-12	-	-	('-', '-')	hp
154	133567	134307	+	gp152 [*Listeria* virus A511]<br><br>nuclease [*Bacillus* phage Chotacabras]	8.0e-100<br><br>3.0e-67	-	-	PF06319.13; MmcB-like; DNA repair protein MmcB-like (99.77/2.30e-17)<br><br>d4qbna_; c.52.1.35 (A:) stNUC {*Salmonella* phage SETP3	MmcB-like protein

				gp124 [*Bacillus* phage W.Ph.]	3.0e-60			[TaxId: 424944]} (96.79/2.90e-02)<br><br>COG1637 NucS; Endonuclease NucS, RecB family [Replication, recombination and repair]. (96.51/7.2e-02)	
155	134300	134803	+	Phage protein [*Enterococcus* phage 156]<br><br>gp153 [*Listeria* virus A511]<br><br>gp130 [*Bacillus* phage W.Ph.]	6.0e-61<br><br>4.0e-38<br><br>6.0e-16	-	-	('-', '-')	hp
156	134814	135680	+	DNA maturase A [*Enterococcus* phage EFP01]<br><br>Phage protein [*Enterococcus* phage 156]<br><br>phage protein [*Staphylococcus* phage MR003]	0.0e+00<br><br>2.0e-91<br><br>7.0e-04	-	-	('-', '-')	hp
157	135792	136340	+	hp	hp	-	-	('-', '-')	hp
158	136353	137201	+	Thioredoxin, phage-associated [*Enterococcus* phage 163]	0.0e+00	-	-	PF10127.10; RlaP; RNA repair pathway DNA polymerase beta family (99.88/6.10e-22)	nucleotidyltransferase

				thioredoxin [*Enterococcus* phage vB_OCPT_Ben]<br><br>thioredoxin [*Enterococcus* phage EFDG1]	0.0e+00<br><br>0.0e+00			COG3541; YcgL; Predicted nucleotidyltransferase [General function prediction only]. (99.75/3.70e-17)<br><br>d1knya2; d.218.1.1 (A:1-125) Kanamycin nucleotidyltransferase (KNTase), N-terminal domain {*Staphylococcus aureus* [TaxId: 1280]} (95.77/5.10e-02)	
159	137201	138928	+	PcfJ-like protein [*Enterococcus* phage PEf771]<br><br>Phage protein [*Enterococcus* phage 156]<br><br>putative PcfJ protein [*Bacillus* phage phiAGATE]	0.0e+00<br><br>2.0e-115<br><br>2.0e-33	PcfJ (412-558)	2.48e-27	PF14284.7; PcfJ; PcfJ-like protein (99.94/1.60e-25)	PcfJ-like protein
160	139203	139898	+	gp165 [*Listeria* virus A511]<br><br>phage protein [*Staphylococcus* phage phiSA039]<br><br>ORF048 [*Staphylococcus* virus Twort]	1.0e-76<br><br>2.0e-70<br><br>1.0e-68	-	-	PF11985.9; DUF3486; Protein of unknown function (DUF3486) (99.56/2.50e-13)<br><br>PF17273.3; DUF5338; Family of unknown function (DUF5338) (97.33/1.20e-03)<br><br>d1iufa1; a.4.1.7 (A:1-75) Ars-binding protein 1, ABP1 {Fission yeast	DUF3486 containing protein

								(*Schizosaccharomyces pombe*) [TaxId: 4896]} (96.74/8.90e-03)	
161	139912	140376	+	Phage protein [*Enterococcus* phage 163]<br><br>gp166 [*Listeria* virus A511]<br><br>structural protein [*Lactobacillus* phage 521B]	1.0e-110<br><br>2.0e-27<br><br>4.0e-17	-	-	PF09629.11; YorP; YorP protein (91.84/7.70e-01)	hp
162	140465	141145	+	hp	hp	-	-	('-', '-')	hp
163	141159	141893	+	hp	hp	-	-	('-', '-')	hp
164	141962	142255	+	hp	hp	-	-	COG5503; RpoEps; DNA-dependent RNA polymerase auxiliary subunit epsilon [Transcription, Defense mechanisms]. (69.09/2.30e+00)	hp
165	142328	144754	+	transposase domain containing protein [*Enterococcus* phage PEf771]<br><br>Phage protein [*Enterococcus* phage 156]<br><br>transposase domain-containing protein	0.0e+00<br><br>3.0e-23<br><br>1.0e-12	-	-	d1rr7a_; a.4.1.14 (A:) Middle operon regulator, Mor {Bacteriophage Mu [TaxId: 10677]} (92.84/5.70e-02)	hp

				[*Staphylococcus* phage Quidividi]					
166	144816	145259	+	minor coat protein [*Enterococcus* phage PEf771]<br><br>minor coat protein [*Enterococcus* phage EFP01]	6.0e-97<br><br>3.0e-91	-	-	('-', '-')	hp
167	145277	145849	+	hp	hp	-	-	('-', '-')	hp
168	146057	146293	+	hp	hp	-	-	PF09925.10; DUF2157; Predicted membrane protein (DUF2157) (94.22/2.60e-01)	hp
169	146355	146603	+	repressor [*Enterococcus* phage EFDG1]<br><br>putative transcriptional regulator [*Enterococcus* phage EF24C]<br><br>putative XRE family transcriptional regulator 1 [*Bacillus* phage BSP10]	8.0e-52<br><br>6.0e-16<br><br>4.0e-07	HTH_XRE (23-77)	1.65e-10	COG5606; COG5606; Predicted DNA-binding protein, XRE-type HTH domain [General function prediction only]. (99.15/2.50e-09)<br><br>d2icta_; a.35.1.3 (A:) Antitoxin HigA {*Escherichia coli* [TaxId: 562]} (99.13/2.80e-09)<br><br>d1b0na2; a.35.1.3 (A:1-68) SinR repressor, DNA-binding domain {*Bacillus subtilis* [TaxId: 1423]} (99.06/1.70e-08)	transcriptional regulator
170	146636	147076	+	dnaJ domain protein [*Enterococcus* phage EFP01]	1.0e-91	-	-	d1q1ha_; a.4.5.41 (A:) Transcription factor E/IIe-alpha, N-terminal domain {*Sulfolobus*	transcriptional regulator

				DnaJ domain protein [*Enterococcus* phage PEf771]<br><br>DnaJ domain protein [*Enterococcus* phage PEf771]	4.0e-49<br><br>6.0e-33			*solfataricus* [TaxId: 2287]} (95.09/1.60e-01)	
171	147228	147551	+	hp	hp	-	-	('-', '-')	hp
172	147568	148056	+	hp	hp	Sigma70_r4 (10-52)	5.23e-04	COG2826; Tra8; Transposase and inactivated derivatives, IS30 family [Mobilome: prophages, transposons]. (98.15/2.90e-05)<br><br>d1tc3c_a.4.1.2 (C:) Transposase tc3a1-65 {Nematode (*Caenorhabditis elegans*) [TaxId: 6239]} (97.59/6.8e-04)	transposase
173	148641	148078	-	HNH endonuclease family protein [*Enterococcus* phage EFRM31]<br><br>DNA endonuclease I-HmuI [*Lactobacillus* phage BH1]<br><br>HNH endonuclease [*Lactobacillus* phage J-1]	5.0e-34<br><br>1.0e-33<br><br>2.0e-33	HNH_3 (65-108)<br><br>NUMOD4 (3-53)	2.98e-10<br><br>9.88e-06	d1u3em1; d.4.1.3 (M:1-105) Intron-encoded homing endonuclease I-HmuI {Bacteriophage SPO1 [TaxId: 10685]} (99.94/2.00e-25)<br><br>PF13392.7; HNH_3; HNH endonuclease (99.32/2.90e-12)<br><br>PF05551.12; zf-His_Me_endon; Zinc-binding loop region of	HNH homing endonuclease

								homing endonuclease (99.29/3.90e-11)	
174	148843	149292	+	hp	hp	-	-	('-', '-')	hp
175	149719	149994	+	Phage protein [*Enterococcus* phage 156]	3.0e-11	-	-	PF06569.12; DUF1128; Protein of unknown function (DUF1128) (90.23/5.90e-01)	hp
176	150118	150246	+	hp	hp	-	-	('-', '-')	hp
177	150307	150495	+	hp	hp	-	-	PF13121.7; DUF3976; Domain of unknown function (DUF3976) (90.93/6.90e-01)	hp
178	150582	150875	+	hp	hp	-	-	('-', '-')	hp
179	151010	151249	+	hp	hp	-	-	('-', '-')	hp
180	151337	151591	+	hp	hp	-	-	('-', '-')	hp
181	151608	151985	+	gp24 [*Brochothrix* phage A9]	6.0e-05	-	-	('-', '-')	hp
182	152005	152178	+	Phage protein [*Enterococcus* phage 156]	7.0e-04	-	-	('-', '-')	hp
183	152358	152597	+	gp24 [*Brochothrix* phage A9]	1.0e-13	-	-	('-', '-')	hp
184	152685	153068	+	hp	hp	-	-	('-', '-')	hp
185	153185	153463	+	hp	hp	-	-	PF08954.12; Trimer_CC; Trimerisation motif (82.59/7.00e+00)	hp
186	153550	153963	+	TreP [*Staphylococcus* phage vB_Sau_Clo6]	2.0e-14	-	-	('-', '-')	hp

				TreP [*Staphylococcus* phage Stab22]<br><br>TreP [*Staphylococcus* phage Stab21]	8.0e-14<br><br>8.0e-14				
187	154086	154355	+	hp	hp	-	-	('-', '-')	hp
188	154981	155136	+	Phage protein [*Enterococcus* phage VPE25]<br><br>Phage protein [*Enterococcus* phage VFW]<br><br>putative exonuclease [*Enterococcus* phage phiEF17H]	4.0e-05<br><br>2.0e-03<br><br>3.0e-03	-	-	('-', '-')	hp
189	155187	155453	+	hp	hp	-	-	d1vqqa2; d.175.1.1 (A:139-327) Penicillin binding protein 2a (PBP2A), middle domain {*Staphylococcus aureus* [TaxId: 1280]} (64.56/4.10e+00)	hp
190	155470	155712	+	hp	hp	-	-	PF16112.6; DUF4830; Domain of unknown function (DUF4830) (95.11/1.30e-02)	hp
191	155729	156049	+	hp	hp	-	-	PF03871.15; RNA_pol_Rpb5_N; RNA polymerase Rpb5, N-terminal domain (88.98/2.60e+00)	hp

192	156123	156263	+	hp	hp	-	-	('-', '-')	hp
193	156353	156496	+	gp16 [*Brochothrix* phage A9]	1.0e-10	-	-	PF13035.7; DUF3896; Protein of unknown function (DUF3896) (87.29/2.50e+00)	hp

Table S3. tRNA and tmRNA genes of the *Enterococcus* phage iF6.

ORF№	Start codon	Stop codon	Strand	Product	Anticodon
1	35400	35328	-	tRNA-Gly	tcc
2	36873	36801	-	tRNA-Ala	tgc
3	36952	36878	-	tRNA-Arg	tct
4	37442	37354	-	tRNA-Ser	tga
5	37624	37538	-	tRNA-Ser	gga
6	38416	38069	-	tmRNA	-
7	38733	38648	-	tRNA-Leu	tag
8	38902	38817	-	tRNA-Leu	taa
9	39150	39080	-	tRNA-His	gtg
10	39932	39859	-	tRNA-Ile	gat
11	40157	40086	-	tRNA-Val	tac
12	40461	40389	-	tRNA-Arg	acg
13	40638	40562	-	tRNA-Lys	ttt
14	40846	40771	-	tRNA-Lys	ctt
15	41111	41038	-	tRNA-Thr	tgt
16	41727	41654	-	tRNA-Phe	gaa
17	41922	41850	-	tRNA-Cys	gca
18	42235	42162	-	tRNA-Trp	cca
19	42518	42445	-	tRNA-Met	cat
20	42798	42725	-	tRNA-Glu	ttc
21	42878	42802	-	tRNA-Asp	gtc
22	44040	43967	-	tRNA-Arg	tcg
23	44202	44130	-	tRNA-Asn	gtt

Table S4. *Enterococcus* phage iF6 and the most closely related phages.

Name	Database accession number	Nucleic sequence length (GenBank)	GC-content, %	CDS (protein-coding)	Number of tRNAs/tmRNA *	BLASTn nucleotide identity to iF6, %**	Proteins shared with iF6 ***		Number of proteins shared with the phage by all of the previous phages of the table above
							number	%	
Enterococcus phage iF6	MT909815.1	156,592	37.1	193	23/1	-	-	-	-
Enterococcus phage 163	CAJDKA010000002.1	150,836	37.0	186	21/1	86	159	42.0	159
Enterococcus phage EFP01	NC_047796.1	155,053	37.0	193	7/0	82	146	37.8	140
Enterococcus phage EFDG1	NC_029009.1	147,589	37.2	192	23/1	80	142	36.7	123
Enterococcus phage PEf771	MN241318.1	151,052	37.0	197	22/1	81	147	37.6	117
Enterococcus phage vB_OCPT_Ben	MN027503.1	151,985	37.1	187	24/1	81	135	35.5	110
Enterococcus phage EfV12-phi1	NC_048087.1	152,770	37.0	191	23/1	82	135	35.2	108
Enterococcus phage 156	LR031359.1	141,133	35.8	209	7/0	40	86	21.4	73

* Determined using ARAGORN v1.2.41;
** Determined using BLASTn compared to iF6 phage (multiplying % coverage by % identity);
*** Determined using GET_HOMOLOGUES (COGtriangles algorithm, -t 0 -C 75).

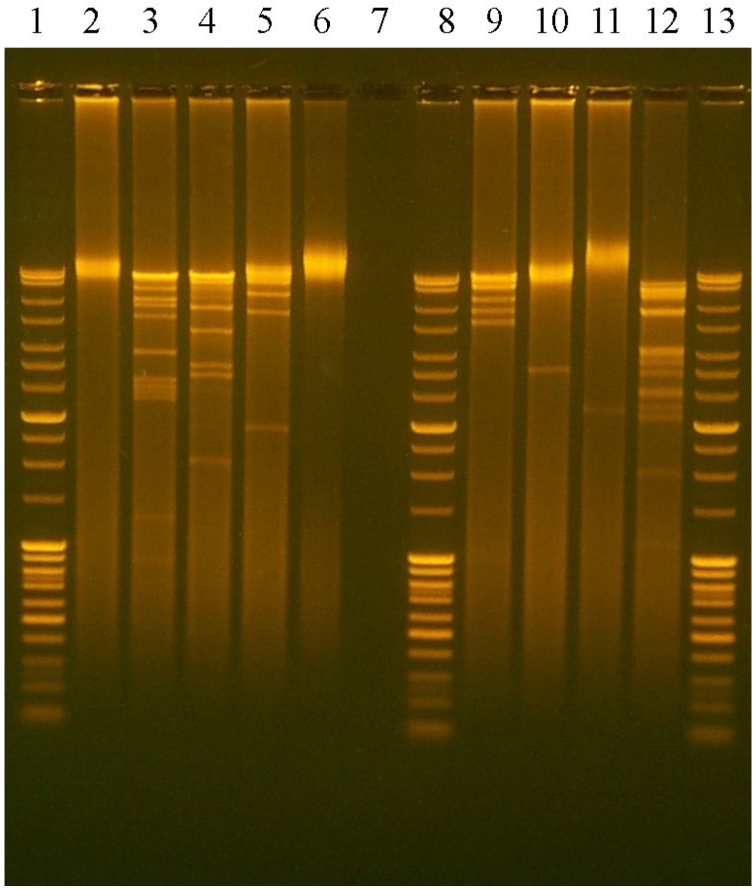

Figure S3. Restriction analysis of iF6 DNA. This is the original gel image used to generate Fig 8 in the main text. Kodak EDAS 290 Gel Documentation System ("Kodak") was used to capture the image. 1, 8 and 13 – molecular weight markers; 6 – intact phage DNA, 7 – the empty slot, 2 – EcoRI, 3 – NsbI, 4 – BlpI, 5 – XhoI, 9 – AfeI, 10 – SalI, 11 – SphI and 12 - KpnI.

Table S5. Primers used to locate the ends of the iF6 genome.

Purpose	Primer name	Primer sequence (5'-3')	№PCR	Template
Determination of the left genome end	RACE-L-iF6_rev1	CATCTAAGTCTTTCAAGTTGTCTAAGTTC	I PCR	Fragment contained the left genome end of iF6 after DNA tailing reaction
	AncherdT16V	GACCACGCGTATCGATGTCGACTTTTTTTTTTTTTTTTV		
	RACE-L-iF6_rev2	TTCTTTGTGTGGTCGTGCTC	II PCR	I PCR product
	Anch	GACCACGCGTATCGATGTCGAC		
Determination of the right genome end	RACE-R-iF6_fwd2	ACTACCAAGGGGGCAATACAG	I PCR	Fragment contained the right genome end of iF6 after DNA tailing reaction
	AncherdT16V	GACCACGCGTATCGATGTCGACTTTTTTTTTTTTTTTTV		
	RACE-R-iF6_fwd3	GGACAACGTACAGACAATTATACCG	II PCR	I PCR product
	Anch	GACCACGCGTATCGATGTCGAC		

Table S6. Primers used for cloning of the iF6 endolysins.

Target gene	GenBank protein number	Vector for cloning	Primer name	Primer sequence (5'-3')
iF6_82	QNL29441.1	pET33b(+)	if6_gp82_NcoI_for	TATATCCATGGCAGGAGAAGTATTTAGTAGT
			if6_gp82_NotI_rev	TATAGCGGCCGCCAATTTGTGAGTTCCACC
		pHUE	if6_gp82_EcoRI_for	TATAGAATTCATGGCAGGCGAAGT
			if6_gp82_KpnI_rev	TATTGGTACCTTACAATTTGTTAGTTCC
iF6_84	QNL29443.1	pET33b(+)	if6_gp84_NcoI_for	TATATCCATGGGTATGAGTAACATTAACATGGAAACC
			if6_gp84_NotI_rev	TATAGCGGCCGCCTTAAATGTACCCCATGCTTTC
		pHUE	if6_gp84_EcoRI_for	TATAGAATTCATGAGTAACATTAACATGGA
			if6_gp84_KpnI_rev	TATAGGTACCTTACTTAAATGTACCC

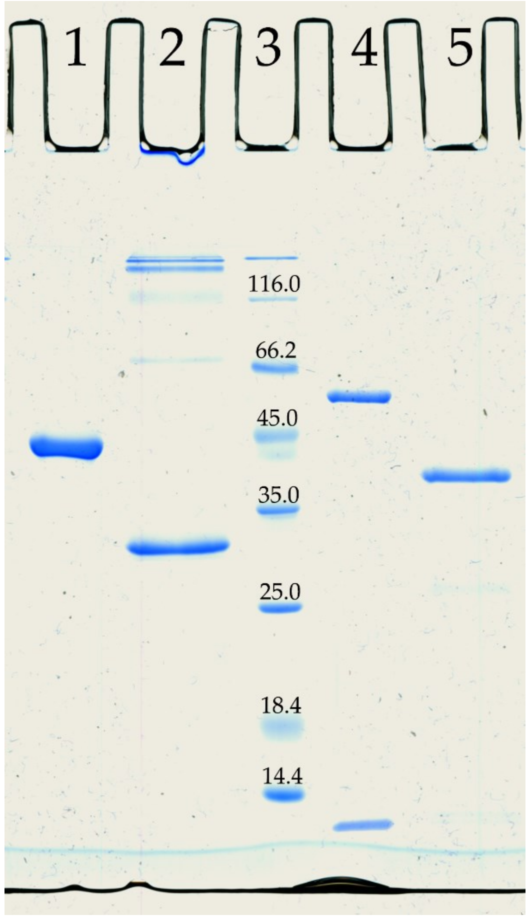

Figure S4. SDS-PAGE analysis of the iF6 phage endolysins. 1 and 2 – purified preparations of Gp82-H (47.6 kDa) and Gp84-H (35.5 kDa), respectively; 3 – Protein marker (Thermo scientific #26610); 4 and 5 – purified preparations of HU-Gp82 (57.4 kDa) and HU-Gp84 (45.5 kDa), respectively. The calculated molecular weights of endolysins shown in brackets. The polyacrylamide (12%) gel was stained with Coomassie Brilliant Blue G-250.
